# Recent progress on vascular endothelial growth factor receptor inhibitors with dual targeting capabilities for tumor therapy

**DOI:** 10.1186/s13045-022-01310-7

**Published:** 2022-07-07

**Authors:** Yun Liu, Yang Li, Yuxi Wang, Congcong Lin, Dan Zhang, Juncheng Chen, Liang Ouyang, Fengbo Wu, Jifa Zhang, Lei Chen

**Affiliations:** 1 Department of Neurology, Joint Research Institution of Altitude Health, West China Hospital, Sichuan University, Chengdu, 610041 Sichuan China; 2 State Key Laboratory of Biotherapy and Cancer Center, West China Hospital of Sichuan University, Chengdu, 610041 Sichuan China; 3 Targeted Tracer Research and Development Laboratory, Frontiers Science Center for Disease-Related Molecular Network, Institute of Respiratory Health, West China Hospital, Sichuan University, Chengdu, 610041 Sichuan China; 4 Department of Pharmacy, West China Hospital, Sichuan University, Chengdu, 610041 Sichuan China

**Keywords:** VEGFR kinase, Antitumor drugs, Dual inhibitor, Antiangiogenesis treatment

## Abstract

Vascular endothelial growth factor receptors (VEGFRs) are a family of receptor protein tyrosine kinases that play an important role in the regulation of tumor-induced angiogenesis. Currently, VEGFR inhibitors have been widely used in the treatment of various tumors. However, current VEGFR inhibitors are limited to a certain extent due to limited clinical efficacy and potential toxicity, which hinder their clinical application. Thus, the development of new strategies to improve the clinical outcomes and minimize the toxic effects of VEGFR inhibitors is required. Given the synergistic effect of VEGFR and other therapies in tumor development and progression, VEGFR dual-target inhibitors are becoming an attractive approach due to their favorable pharmacodynamics, low toxicity, and anti-resistant effects. This perspective provides an overview of the development of VEGFR dual-target inhibitors from multiple aspects, including rational target combinations, drug discovery strategies, structure–activity relationships and future directions.

## Introduction

**Fig. 1:**
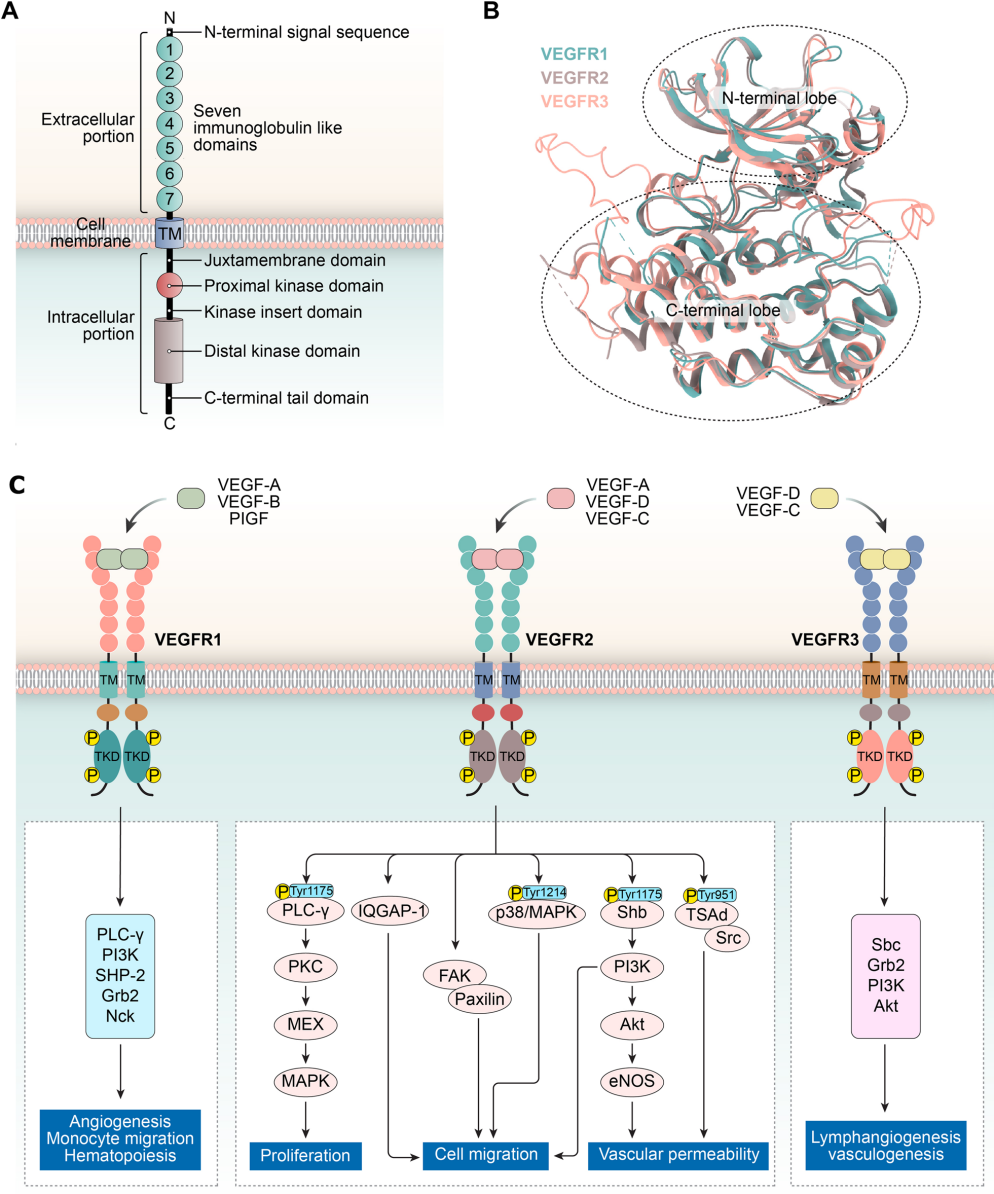
**A** Schematic representation of the VEGFR protein domain structure; **B** the overlap of crystal structures of VEGFR3 (pink), VEGFR2 (PDB ID: 3WZE, brown) and VEGFR1 (PDB ID: 3HNG, blue). The structure of VEGFR1 and VEGFR2 was utilized to construct the homology model of human VEGFR3; **C** mechanisms of tumor angiogenesis regulated by VEGFR signaling

Abnormal angiogenesis can be considered an essential prerequisite for tumor progression and metastasis. Existing pieces of evidence have demonstrated that many extracellular, cell surface and intracellular molecules can directly or indirectly regulate angiogenesis [1, 2]. In particular, vascular endothelial growth factors (VEGFs) and their interaction with membrane receptors are of great significance during angiogenesis. In mammals, VEGF isoforms [VEGF-A, B, C, D and placental growth factor (PIGF)] are encoded by VEGF-related genes and interact specifically with the VEGF receptors (VEGFRs) family of VEGFR-1/Flt-1, VEGFR2/KDR and VEGFR-3/Flt-4 [3, 4]. These receptors share a high degree of structural similarity, but differ in activation mode, signal transduction and biological functions [5]. Table 1 summarizes the specific ligands, main functions and distinct domains of these receptors. Briefly, VEGFR1, VEGFR2 and VEGFR3 are essential for the development of hematopoietic cells, vascular endothelial cells and lymphatic endothelial cells, respectively. Nevertheless, VEGFR3 and its ligands play critical roles in lymphangiogenesis and the spread of tumor cells to regional lymph nodes [6, 7]. Structurally, VEGFRs consist of an extracellular part consisting of an extracellular ligand-binding domain (ECD) with seven immunoglobulin-like domains (IgD), a single transmembrane domain (TM), a juxtamembrane domain (JMD), a tyrosine kinase domain (TKD) with an insert of approximately 80 residues, and a carboxyl terminus (Fig. 1A, B) [8]. The activation of VEGFRs can be mediated by ligand binding. Subsequently, ligand-induced conformational changes in the VEGFRs intracellular domain promote receptor dimerization, leading to the autophosphorylation of specific tyrosine residues and the activation of several downstream enzymatic pathways, including p38/MAPK, RAS/RAF/MEK/ERK and PI3K/AKT/mTOR (Fig. 1C). At the same time, some receptors undergo internalization and form endosomes. In the early stages of internalization, receptors still exist as a membrane protein component of endosomes, and this receptor compartment, composed of microtubules and vacuoles, is widely distributed in the cytoplasm. During the transit of receptor across the endosomal membrane, the ligand–receptor complexes remain intact and the kinase function of the receptors remains activated. Finally, membrane fragments containing ligand–receptor complexes are squeezed into the lumen of the endosome as small vesicles, thus forming multi-vesicular endosomes. As a result, ligand–receptor complexes on the plasma membrane reach the lumen of the endosome and are widely distributed throughout the cytoplasm, along with other contents of the lumen. This process attenuates the continuous stimulation of growth factors at the cell surface and allows for a broad distribution of ligand–receptor complexes within the cytoplasm [9]. Importantly, it has been previously shown that VEGF could induce the internalization of VEGFR1 and VEGFR2 [10].

**Table 1:** Unique characteristics of the VEGFR family

Receptor	VEGFR1	VEGFR2	VEGFR3
Protein size	180–185 kDa	210–230 kDa	195 kDa
Ligands	VEGF-A, VEGF-B and PIGF	VEGF-A, VEGF-C and VEGF-D	VEGF-C and VEGF-D
Functions	A negative regulator of angiogenesis, vasculogenesis and monocyte/macrophage motility	Vasculogenesis, angiogenesis, vascular permeability and endothelial cell motility and survival	Vascular and lymphatic development and maintenance
Full length	1338	1356	1363
Signal peptide	1–26	1–19	1–24
Receptor chain	27–1338	20–1356	25–1363
ECD	27–758	20–764	25–775
IgD1	32–123	46–110	30–127
IgD2	151–214	141–207	151–213
IgD3	230–327	224–320	219–326
IgD4	335–421	328–414	331–415
IgD5	428–553	421–548	422–552
IgD6	556–654	551–660	555–671
IgD7	661–747	667–753	678–764
TM	759–780	765–785	776–796
Cytoplasmic domain	781–1338	786–1356	797–1363

The dysfunctional VEGF-VEGFR signal axis is widely involved in human diseases, especially tumors. Inhibitors targeting VEGF signaling, including monoclonal antibodies targeting VEGF and small molecules targeting VEGFR, have shown treatment efficacy for different types of solid tumors. Specifically, bevacizumab, as a recombinant humanized monoclonal antibody targeting VEGF, exerts beneficial clinical effects. However, the main issues of anti-VEGF monoclonal antibodies are the high immunogenicity, high cost and low stability. Furthermore, the clinical application of anti-VEGF monoclonal antibodies is severely limited by considerable side effects associated with the inhibition of physiological angiogenesis, which is one of the most common side effects of antiangiogenic therapies. Currently, targeting tumor angiogenesis via inhibiting VEGFRs has become a successful strategy for oncotherapy [11]. To date, more than 340 clinical trials related to VEGFR inhibitors have been retrieved from the Web site of www.ClinicalTrials.gov. Specifically, several VEGFR inhibitors have been approved for clinical use, and their efficacy results are summarized in Table 2. Notably, VEGFR inhibitors could be divided into three classifications: type I inhibitors, type II inhibitors and type III inhibitors [12]. Type I inhibitors [*e.g.*, sunitinib (**2**), pazopanib (**3**), vandetanib (**4**), axitinib (**5**), ponatinib (**9**) and motesanib (**11**)], also known as ATP competitive inhibitors, could generate hydrophobic interactions with the adenine region and form one to three hydrogen bonds with the surrounding residues at the active site of the receptor, thereby competing for binding to the active “DFG-in” conformation in the ATP-binding pocket [13]. Type II inhibitors [e.g., sorafenib (**1**), carbozantinib (**6**), lenvatinib (**7**), regorafenib (**8**) and lucitanib (**10**)] are characterized by binding to the inactive “DFG-out” conformation of the kinase and occupying a hydrophobic pocket adjacent to the ATP-binding site [14, 15]. Type III inhibitors [e.g., vatalanib (**12**)], or called covalent inhibitors, could exert their pharmacological functions through irreversibly binding to cysteines at specific sites on the kinases [16]. So far, numerous approved VEGFR inhibitors are type I inhibitors, which target the ATP-binding pocket. Based on the X-ray crystal structure of VEGFR2, several type I inhibitors have been reported to exert regulatory effects on tumor suppression. However, several studies have demonstrated that type II inhibitors possess certain advantages over type I inhibitors, including improved potency and selectivity [17, 18]. Structurally, the extension into the less conservative allosteric hydrophobic back pocket facilitates the affinity and selectivity of the type II inhibitors [19]. In addition, covalent enzyme inhibitors have been widely applied as therapeutic agents [20]. Generally speaking, most of these inhibitors can achieve continuous amelioration and even cure some tumor patients. However, their clinical application is limited by therapeutic resistance, limited efficacy and off-target toxicity [6]. Firstly, the mechanisms of resistance to VEGFR inhibitors are classified into the following sections: (i) activation of alternative pro-angiogenic signaling pathways; (ii) recruitment of local and distal stromal cells; and (iii) alternative modes of tumor vascularization (e.g., hypoxia). Secondly, due to similarities in the kinase domains of VEGFR and other receptors, these inhibitors showed cross-inhibitory activities against other targets such as PDGFR, c-KIT, and FLT3, leading to possible off-target effects. Several clinical toxic effects of VEGFR inhibitors have been investigated, such as hypertension, proteinuria, hypothyroidism, leukoencephalopathy syndrome and arterial thrombosis. Finally, accumulating pieces of evidence have confirmed that several VEGFR inhibitors have generally failed to reveal remarkable overall efficacy in the clinic [21, 22]. Therefore, clinical strategies to overcome these drawbacks, such as combination therapy, need to be well concerned [23, 24].

**Table 2:** Summary of clinically approved VEGFR inhibitors

Drugs	Chemical structure	Target	Tumor types	Released date	Type	Ref
Sorafenib (1)		VEGFR2/3, PDGFRβ, c-Kit, BRAF	Advances renal cell carcinoma, hepatocellular carcinoma	2007	II	[25]
Sunitinib (2)		VEGFR1/2, PDGFRβ, FLT3	Renal cell carcinoma, gastrointestinal stromal tumors	2006	I	[26]
Pazopanib (3)		VEGFR2, PDGFRβ, c-Kit	Hepatocellular carcinoma	2012	I	[27]
Vandetanib (4)		VEGFR2/3, EGFR, RET	Late-stage metastatic medullary thyroid tumor	2011	I	[28]
Axitinib (5)		VEGFR1/2, c-Kit	Renal cell carcinoma	2012	I	[29]
Carbozantinib (6)		VEGFR2, c-Met, RET	Thyroid tumor	2012	II	[30]
Lenvatinib (7)		VEGFR1/2/3	Thyroid tumor	2015	II	[31]
Regorafenib (8)		VEGFR1/2/3, PDGFRβ, c-Kit, BRAF	Colorectal tumor, advanced GI stromal cancer, hepatocellular carcinoma	2015	II	[32]
Ponatinib (9)		Abl, PDGFRα, VEGFR2, FGFR1, Src	Chronic myeloid leukemia, acute lymphoblastic leukemia	2012	I	[33]
Lucitanib (10)		VEGFR1/2/3, FGFR-1, FGFR-2	Metastatic breast tumor	Phase II clinical trials	II	[34]
Motesanib (11)		VEGFR1/2/3	Non-small-cell lung tumor	Phase III clinical trials	I	[35]
Vatalanib (12)		VEGFR1/2/3, PDGFRβ, c-Kit	Colorectal tumor	Phase III clinical trials	III	[36]

Combination therapy in human tumors not only increases the potency, but also reduces potential adverse events [37]. Since the therapeutic efficacy in tumors and relevant biological functions of these enzymes have been revealed, it is considered that a combination of them with VEGFR inhibitors (e.g., epigenetic agents, immunotherapeutic drugs and other RTK inhibitors) can be promising in antitumor treatment. However, it should be cautious that the complicated doses/schedule, dubious pharmacokinetic/pharmacodynamic profile and potential adverse events require in-depth explorations [38, 39]. As an alternative strategy to combination therapy, dual-target or multi-target drugs are characterized by reduced risk of adverse drug–drug interactions (DDIs), better pharmacokinetic (PK) profiles and guaranteed safety [40]. Based on these concepts, VEGFR dual-target inhibitors are emerging as an attractive approach.

Given the synergistic effect of VEGFR and other therapies in tumor development and progression, the identification of novel VEGFR dual-target inhibitors may provide an effective strategy for clinical practice. From this perspective, the research progress of dual-target VEGFR inhibitors is summarized, focusing on the rational targets selection, structure–activity relationships (SARs) and pharmacological activities of dual-target VEGFR inhibitors.

### VEGFR2 as a therapeutic target

As mentioned above, each VEGFR family has unique characteristics. Among them, VEGFR2 has been identified as a promising tumor therapy target [41]. Accumulating pieces of evidence have confirmed that the abnormal expression of VEGFR2 in neovascular tumor endothelial cells is closely linked to the occurrence and development of multiple types of tumors [42, 43]. By blocking angiogenesis and lymphangiogenesis, all VEGFR2 inhibitors offer varying degrees of clinical benefit against different types of tumors, although most of them lack specificity [44].

### Research status of VEGFR2 inhibitors

**Fig. 2:**
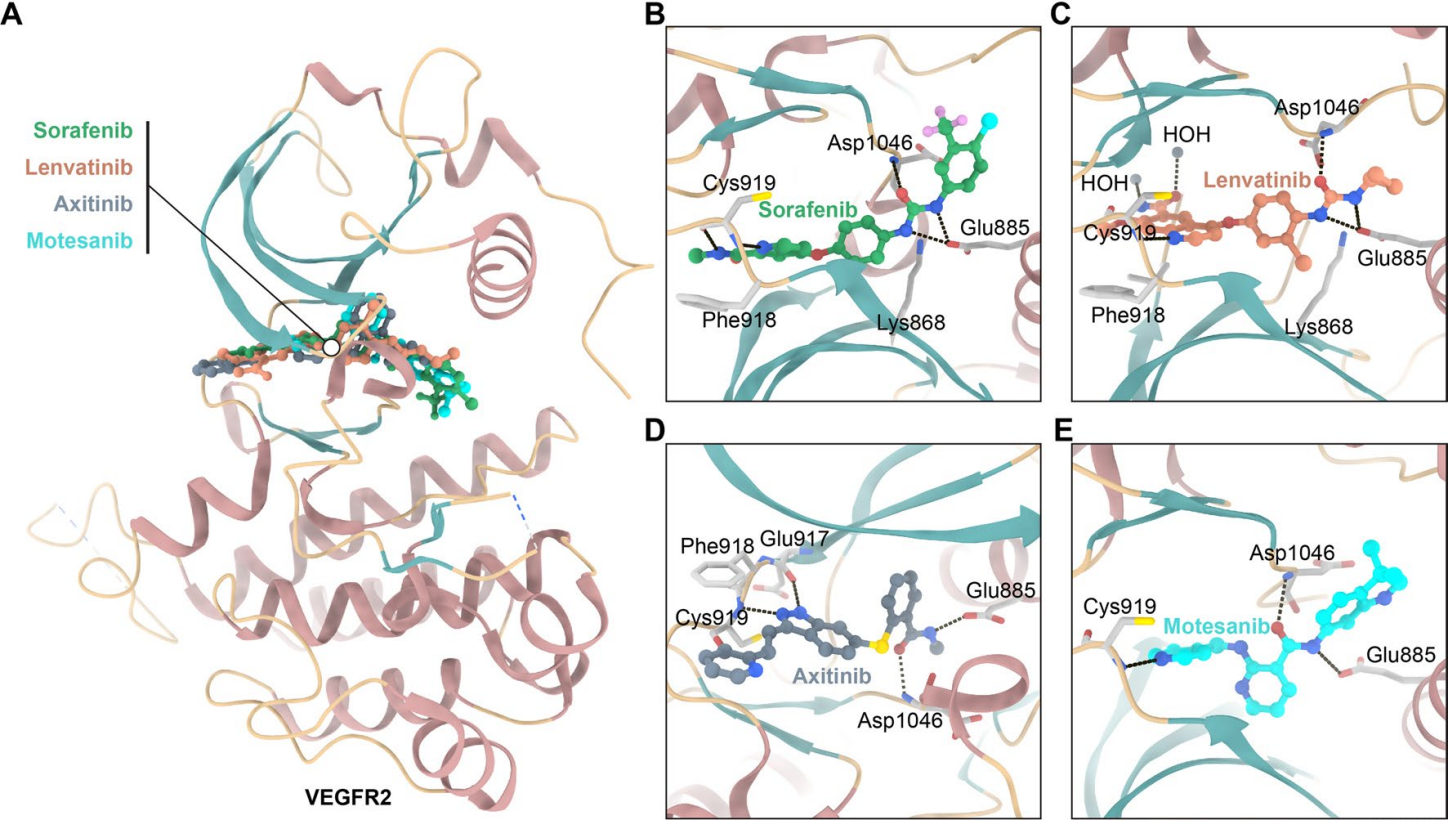
Crystal structure of the FDA-approved VEGFR inhibitors—VEGFR2 complex. The corresponding PDB codes are 3WZE (sorafenib), 3WZD (lenvatinib), 4AGC (axitinib) and 3EFL (motesanib). Hydrogen binding (black) interactions are shown as dashed lines. **A** The overlap of co-crystal structures of VEGFR2 with sorafenib (green), lenvatinib (orange), axitinib (gray) and motesanib (blue); **B–E** Binding modes of VEGFR2 with sorafenib, lenvatinib, axitinib and motesanib

In the pharmaceutical field, the discovery and development of highly potent VEGFR2 inhibitors have always been a research hotspot. Co-crystal structures of VEGFR2 complexed with FDA-approved inhibitors reveal structural information for the structure-based design of VEGFR2 inhibitors. Structurally, the catalytic domain of VEGFR2, as a bi-lobed structure with a small N-lobe and a large C-lobe, plays a key role in the inhibitory potency of these inhibitors. Specifically, the active site of VEGFR2 consists of the following subregions: hydrophobic region I (encapsulated by the residues Leu840, Phe918 and Gly922), hydrophobic region II (encapsulated by the residues Leu889, Ile892, Val898 and Ile1044) and one linker region (encapsulated by the residues Ala866, Val914, Leu1035 and Cys1045) [45]. As shown in Fig. 2, the co-crystal structures of VEGFR2 in complex partial FDA-approved inhibitors revealed that these inhibitors, although highly structurally diverse, share conclusive pharmacophoric characteristics. Firstly, a flat heteroaromatic ring system of the primary skeleton that adopts the active site through the formation of a key hydrogen bond with Cys residue, and the essential residue in the catalytic ATP-binding pocket. Thus, at least one hydrogen bond acceptor should be included in this flat system (N atoms are preferred, followed by O atoms). Secondly, the linker region between the ATP-binding pocket and the DFG domain of the enzyme is occupied by a central aryl ring or spacer. Thirdly, functional groups such as amides or ureas, which are pharmacophores, form two hydrogen bonds with the side chain of Glu885 residue in the C-helix and the backbone NH of Asp1046 residue in the DFG motif, respectively. Fourthly, the terminal hydrophobic moiety of these inhibitors forms the hydrophobic interaction with the allosteric hydrophobic pocket [46, 47]. Based on these findings, several inhibitors of VEGFR2 containing different cores have been reported to suppress tumor growth.

### Synergistic effects of VEGFR2 inhibitors and other antitumor agents

Through preclinical or clinical evaluation, the antitumor potency of VEGFR2 inhibitors combined with other antitumor drugs has been extensively identified. Existing pieces of evidence have shown that the combination of other RTK inhibitors and VEGFR inhibitors exerted a favorable clinical perspective [48, 49]. Firstly, the combination therapy of the dual epidermal growth factor receptor (EGFR) inhibitor cetuximab and VEGFR inhibitor sorafenib prominently enhanced the clinical benefit of KRAS-mutated metastatic colorectal tumor in phase II clinical trial (NCT00326495). Secondly, aberrant expression of VEGFR and genetic alteration of fibroblast growth factor receptor (FGFR) have been reported to be involved in the development of solid tumors, synergistically promoting angiogenesis and fibrosis. Pieces of clinical evidence have suggested that lucitanib, as a dual VEGFR–FGFR inhibitor, exhibited significant inhibitory activities against solid tumors (NCT01283945). Finally, available pieces of evidence have demonstrated that the synergistic collaboration of VEGFR and c-Met promotes the process of angiogenesis and the development of multiple types of tumors [50]. Clinical evidence has also demonstrated the enhanced therapeutic efficacy of c-Met inhibitor tivantinib in combination with the VEGFR inhibitor pazopanib in advanced solid tumors (NCT01468922).

Rapidly accelerating fibrosarcoma (RAF) homologs, as serine threonine kinases, are of significance in regulating the RAS-RAF-MEK-ERK pathway, which have been highlighted as a potent antitumor target. Among mammalian genes, RAF homologs are encoded by three independent genes, including ARAF, BRAF and CRAF. Among them, BRAF presents the most remarkable reactivity of the others and can be activated by mutation in tumor cells. To date, mutations at valine 600 (V600D, V600E, V600K and V600R) have been detected in different types of tumors. Compared with wild-type BRAF, BRAF^V600E^, the most common mutation, can significantly improve (approximately 600-folds) the kinase activity and ultimately promote the development of tumors [51, 52]. Recently, multiple studies have demonstrated that BRAF and VEGFR2 exert a synergistic effect on the development of tumors, and thus, combination therapy involving VEGFR2 inhibitors and BRAF inhibitors has been identified as a promising strategy for the treatment of tumors [53, 54]. Despite the multi-target inhibitors **1** and **8**, RAF265, a potent RAF/VEGFR2 dual inhibitor, has been identified and successfully applied to clinical treatment (NCT00304525) [55].

HDAC isozymes can be utilized as promising therapeutic targets for tumors. So far, accumulating pieces of preclinical and clinical evidence have shown that the combination of HDAC inhibitors with VEGFR inhibitors is promising in antitumor therapy. Particularly, in vitro and in vivo pieces of evidence proved the synergistic effects of compound **3** and diverse HDAC inhibitors for drug resistance reversal and enhanced antitumor efficacy [56, 57]. Moreover, a phase I trial evaluated the application of an HDAC inhibitor, SAHA, in combination with compound **3** in patients with mutant *TP53*, particularly in patients with metastatic sarcoma or metastatic colorectal tumor, and exerted considerable toxicities [58]. In another phase I trial, combination therapy with compound **3** and the HDAC inhibitor abexinostat demonstrated that HDAC inhibition could promote response and reverse resistance to compound **3** in patients with renal cell carcinoma and other solid tumor malignancies [59]. Severing as key components of cytoskeletons in eukaryotic cells, microtubules play an important role in a number of cellular functions. Due to their specific functions in crucial cellular processes, microtubules have been highlighted as potent antitumor targets [60]. In clinical trials, the combination of bevacizumab (anti-VEGF monoclonal antibody) and paclitaxel (microtubule-targeting agents) significantly increased the antitumor responses [61].

The estrogen receptor alpha (ERα) is utilized as a promising therapeutic target for breast tumor therapy, and VEGFRs play an important role in the development of breast tumors. In 2010, Roshani et al*.* reported the therapeutic effect of combining tamoxifen, a selective estrogen receptor modulator (SERM), and brivanib in human breast cancer cells. In vitro and in vivo pieces of evidence supported the role of combination therapy involving SERM and VEGFR2 inhibitors in improving therapeutic efficacy, as well as inhibiting the growth of SERM-resistant tumors [62].

Previous studies have proven the vital role of hypoxia-mediated abnormal expression of PIM1 in antiangiogenic drug resistance [63]. In 2018, Andrea L et al*.* demonstrated that a combination of PIM1 kinase inhibitors with antiangiogenic drugs can be promising in the treatment of solid tumors [64]. In vitro and in vivo studies showed that the synergy of PIM1 inhibitors and VEGF-targeting agents led to reduced proliferation, lessened tumor vasculature and decreased metastasis [65].

Collectively, these studies demonstrated a significant therapeutic advantage for VEGFR inhibitor-based combination therapies. Specifically, they not only present favorable potency, but may also reverse drug resistance. However, drug combination therapies are limited by the complicated doses/schedule, dubious pharmacokinetic/pharmacodynamic profile and potential adverse events. Encouragingly, the cognition of synergetic efficacy of these drug combinations by clinical investigations and phenotype screenings facilitated the rational combinations of numerous targets to identify dual-target VEGFR inhibitors.

### Design approaches for dual-target VEGFR inhibitors

Dual-target strategies possess several advantages over single-target drugs and drug combination therapy. Firstly, dual-target drugs not only retain most of the advantages of combination therapy, but also partially overcome the shortcomings of combination therapy. Specifically, due to one integrated molecule, dual-target drugs possess no or lower risks of drug–drug interactions, lower adverse reactions, more effortlessly predictable PK profiles, lower incidence of target-based resistance and higher patient compliance [66].

Rational target combinations have been found to play a key role in the clinical successes of dual-target drugs, ultimately facilitating the development of dual-target VEGFR inhibitors [67]. To date, great efforts have been made to identify dual-target drugs. In general, design strategies such as drug repurposing, pharmacophore-based combination and computational approaches are frequently used for dual-target drug discovery [68]. Specifically, drug repurposing is the application of conventional agents to novel therapeutic fields and is characterized by exerting a shorter development process [69]. Additionally, most dual-target VEGFR inhibitors are identified via the pharmacophore-based approach. This approach is characterized by integrating the potency of two selective inhibitors into a single molecule and is carried out by linking or merging the pivotal pharmacophores of selected maternal inhibitors. A pharmacophore-linked method is a simple approach through directly connecting pharmacophores or via a conjugate linker. However, the pharmacophore-linked molecules also possibly suffer from high molecular weight, low absorption and poor PK properties. In addition, an inappropriate linker would hamper the interaction of the ligand moiety with the target protein [70]. Similar to a hybrid design, the pharmacophore-merged strategy is an approach to obtaining new chemical structures by maximizing the overlapping level of pharmacophores, resulting in smaller molecular weight, simple skeleton and better physicochemical properties than those of the parent drugs [71]. However, any alterations in the structure of the parent drug may result in vital changes in biological activities [72]. Thus, it is important to determine the mutual pharmacophores of both VEGFR and other targets before designing dual-target VEGFR inhibitors. Undoubtedly, drug repurposing and pharmacophore-based approaches are essential for the discovery of dual-target drugs. However, the application of these strategies is based on known small molecules, thus leading to the poor structural diversity of dual-target VEGFR inhibitors. Nowadays, computational approaches have been successfully applied to identify dual-target drugs with desired activity profiles, including ligand/structure-based drug design, in silico screening and data mining [73]. Specifically, these approaches are capable of predicting novel targets of reported ligands and are also of significance for the identification or optimization of novel ligands for desired targets. In particular, several dual-target VEGFR inhibitors, containing novel scaffolds, are identified via molecule docking, pharmacophore studies and binding pocket similarity search, showing better therapeutic efficacy for tumors.

## Dual inhibitors of VEGFR2 and other tumor-associated targets

### Dual VEGFR2–EGFR inhibitors

**Fig. 3:**
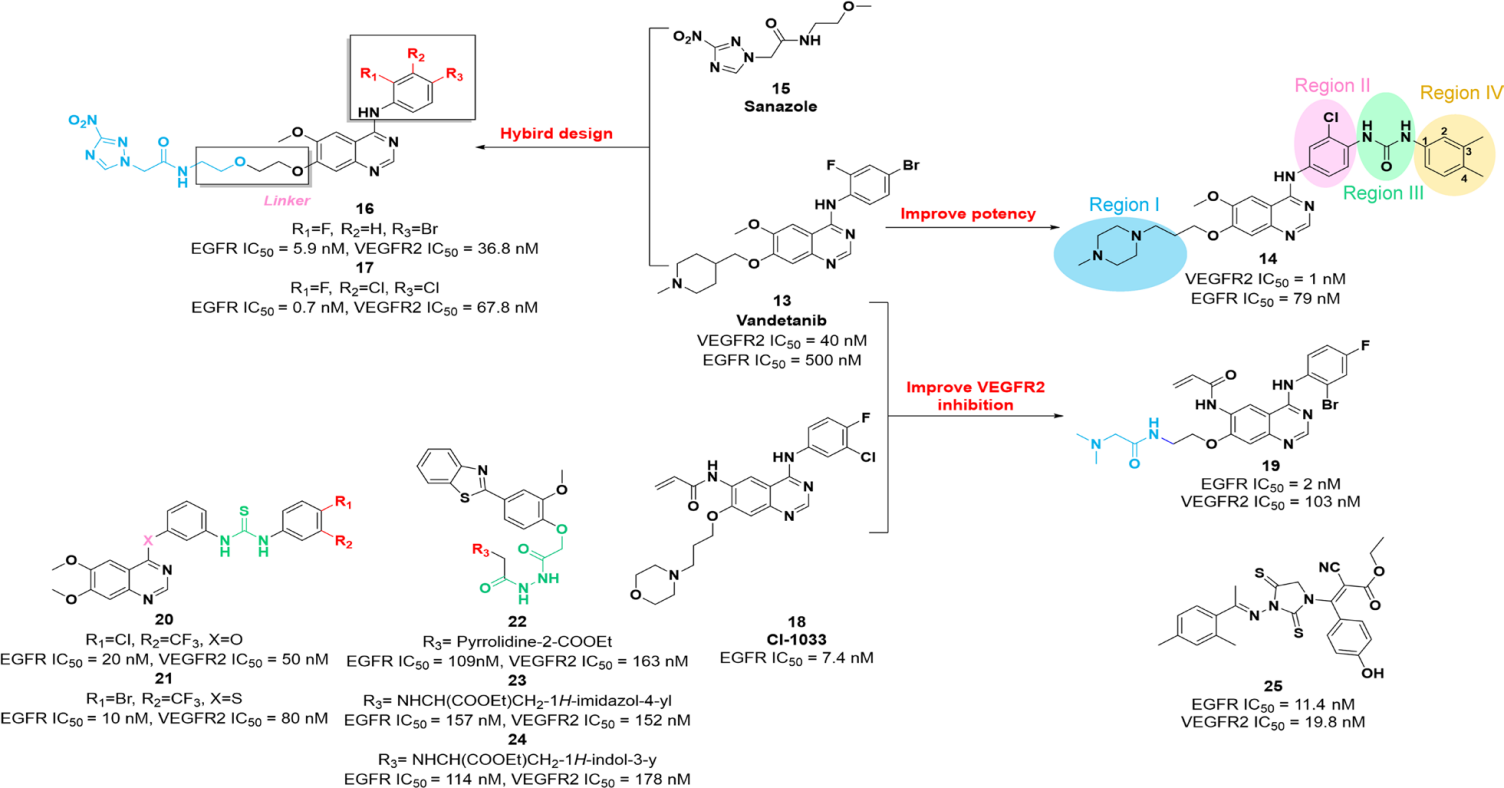
Chemical structures of dual VEGFR2–EGFR inhibitors **14**, **16**, **17** and **19–25** and their inhibitory activities against VEGFR2 and EGFR

Accumulating pieces of evidence have confirmed that VEGFR2 is closely related to EGFR and is involved in the development of multiple types of tumors [74, 75]. Thus, the combination of VEGFR2 inhibitors and EGFR inhibitors can be promising in antitumor treatment. Accordingly, several VEGFR2–EGFR dual inhibitors have been reported to exert promising effects on tumor suppression. Chemical structure, in vitro potency and optimization of dual VEGFR–EGFR inhibitors are illustrated in Fig. 3.

Due to their suitable physicochemical properties, diaryl urea and amide groups have been widely used in VEGFR2 inhibitors design. In 2017, the analogue **14** (Fig. 3) was obtained by optimizing the side chain and introducing chlorine within the central core of compound **13** (vandetanib). It shows a remarkable potency against VEGFR2 and EGFR with IC_50_ values of 1 nM and 79 nM, respectively. Compared with parent compound **13**, **14** exerts superior inhibitory activities against HT-29 and MCF-7 cells (IC_50_ = 1.76 μM and 7.28 μM, respectively). Preliminary SAR studies showed that (i) compounds containing the 4-methylpiperazine group in the region I position exerted higher potency against VEGFR2 and EGFR; (ii) the introduction of chlorine in the region II position could facilitate the kinases inhibition of both VEGFR2 and EGFR; (iii) the introduction of the diaryl urea group at region III is beneficial to improve the potency; and (iv) benzene ring with a methyl group at C-3 and C-4 positions in region IV could improve potency.

More and more pieces of evidence have confirmed that hypoxia is closely linked to the occurrence and development of multiple types of tumors. Additionally, hypoxia is the main cause of therapeutic resistance, especially in radiotherapy. Presently, owing to the significance indicated in tumor progression and drug resistance, molecules in hypoxia-driven pathways are considered as potential therapeutic targets for tumors [76]. Based on these studies, a series of hypoxia-targeted EGFR/VEGFR2 dual inhibitors containing 3-nitro-1,2,4-triazole core is prepared by Wei et al*.* [77]. Compared with **13**, most of these compounds exert superior potency against EGFR, with IC_50_ values in the low nanomolar range. Moreover, they also show good-to-moderate inhibitory activities against VEGFR2 with IC_50_ values in the concentration range between 36.8 nM and 4.09 μM. Among these compounds, compounds **16** and **17** (Fig. 3) showed the most remarkable inhibitory activity against VEGFR2 and EGFR. Furthermore, in vitro and in vivo evidence proved that compound **16** has superior therapeutic efficacy, target selectivity and acceptable tolerance. Further SAR studies showed that the length of the linker could dramatically influence the potency of target compounds against EGFR, and bulky and heavy halogen-substituted benzene contributed to the improvement in inhibitory activities against VEGFR2.

In 2018, Bang et al. identified compound **19** based on the structures of molecule **13** and second-generation EGFR inhibitor **18** (CI-1033), which is a powerful VEGFR2/EGFR dual inhibitor [78]. In vitro enzymatic inhibition assay showed that **19** exerts potent inhibitory potency against both VEGFR2 and EGFR with the IC_50_ values of 103 nM and 2 nM, respectively. Furthermore, **19** showed remarkable inhibitory activities against EGFRT790M and EGFRT790M/L858R mutants with IC_50_ values of 11 nM and 3 nM, respectively. In 2017, compounds **20** and **21** were developed based on the structure of **1**, which present selective, cell active and potent potency against VEGFR2 (IC_50_ = 50 nM and 80 nM, respectively) and EGFR (IC_50_ = 20 nM and 10 nM, respectively) in vitro*.* In in vivo models, **20** and **21** present a competitive tumor suppression role than molecule **1** [79]. Similarly, compounds **22**, **23** and **24**, as derivatives of **1**, were also identified by Eman et al. These molecules exert potential inhibitory activities against VEGFR2 (IC_50_ = 163 nM, 152 nM and 178 nM, respectively) and EGFR (IC_50_ = 109 nM, 157 nM and 114 nM, respectively). Furthermore, these compounds showed low micromolar potency against different types of tumor cells in vitro [80]. In 2021, Mourad et al*.* identified a series of novel VEGFR2–EGFR dual inhibitors containing 2-thioxoimidazolidin-4-one scaffold [81]. Among these compounds, compound **25** has a promising potency for VEGFR2 and EGFR (IC_50_ = 19.8 nM and 11.4 nM, respectively), and stronger antitumor effects on human breast cancer cell lines MCF-7 compared with that of molecule **1** and EGFR inhibitor erlotinib. Furthermore, **25** promotes cell apoptosis and the prolongation of cell cycle progression in the G2/M-phase against MCF-7 cells.

### Dual VEGFR2–FGFR inhibitors

**Fig. 4:**
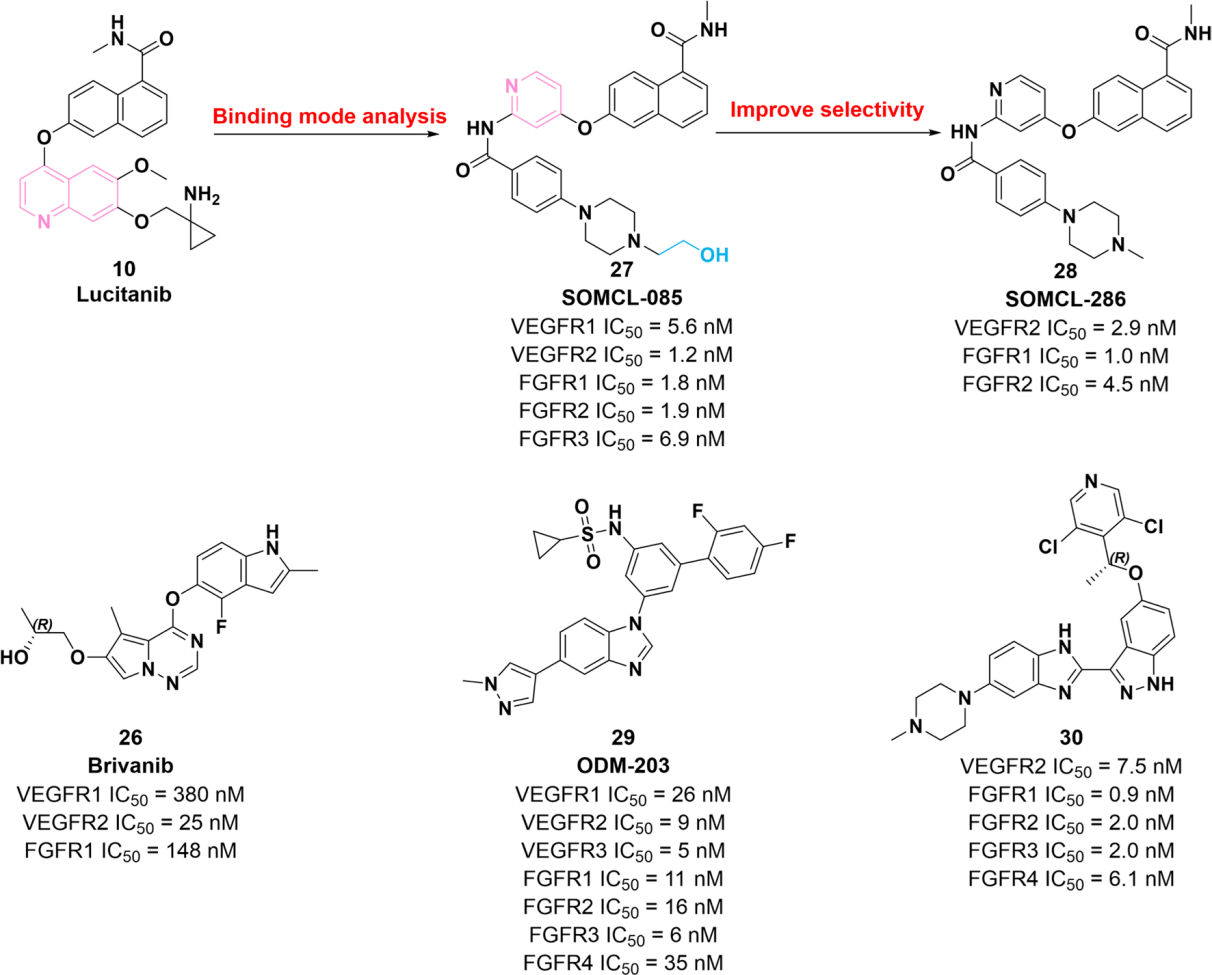
Chemical structures of dual VEGFR2–FGFR inhibitors **26**–**30** and their inhibitory activities against VEGFR2 and FGFR

Accumulating pieces of evidence have confirmed that the binding of VEGF ad VEGFR and the binding of FGF2 and FGFR are synergistically involved in angiogenesis and fiber formation, thereby mediating the development of tumors [82]. Up to now, several dual inhibitors of VEGFR and FGFR containing different cores have been reported to suppress tumor growth [83]. Their chemical structure, in vitro and in vivo potency, and optimization are illustrated in Fig. 4.

In 2006, compound **26** (brivanib) was identified as a promising inhibitor of VEGFR2 and FGFR by Bhide et al*.* [84]. **26** exerts remarkable inhibitory potency against VEGFR1, VEGFR2 and FGFR1 with the IC_50_ values of 380 nM, 25 nM and 148 nM, respectively. Preliminary SAR studies showed that (i) the introduction of methyl group at the 5-position of the pyrrole[2,1-*f*][1,2,4]triazine ring improves the inhibitory activity against VEGFR2; (ii) the substitution of indole ring at the 4-position of fluorine atom is beneficial to improve the potency against VEGFR2; (iii) the superior enzyme potency is attributed to the replacement of ester group at 6-position with an ether group; and (iv) CYP3A4 can be strongly suppressed through introducing the amino side chain. However, **26** is limited by the poor oral bioavailability and low absorption. Thus, an ester pro-drug of **26** is prepared by Cai et al*.* by introducing L-alanine in the side chain of molecule **26** [85]. Preclinical studies have demonstrated that **26** exerts a significant antiangiogenic efficacy through simultaneously blocking FGF and VEGF pathways [86]. Until now, there have been several clinical trials of **26** in the treatment of different types of tumors (NCT04395612, NCT03895788, NCT03516071 and NCT04212221).

Based on the study of binding mode of **10** and FGFR, compound **27** (SOMCL-085) is further discovered to present powerful potency against FGFR1, FGFR2, FGFR3, VEGFR1, VEGFR2, PDGFRα and PDGFRβ (IC_50_ = 1.8 nM, 1.9 nM, 6.9 nM, 5.6 nM, 1.2 nM, 22.6 nM and 7.8 nM, respectively). Specifically, **27** is obtained through opening the quinoline fragment of 10 and introducing the amide as a hydrogen bond acceptor and donor. In the following in vitro and in vivo assays, compound **27** is determined to present a considerable selectivity profile and antiproliferative activities [87]. In 2018, Wei et al*.* identified compound **28** (SOMCL-286) based on the structure of molecule **27**, which is a potent VEGFR2/FGFR dual inhibitor [88]. In vitro enzymatic inhibition assay showed that **28** exerts potent inhibitory potency against VEGFR2, FGFR1 and FGFR2 with the IC_50_ value of 2.9 nM, 1.0 nM and 4.5 nM, respectively. Nevertheless, **28** presents superior selectivity for VEGFR2 and FGFR compared with that of molecule **27**. Therefore, **28** theoretically exerts superior curative effect and low toxicity. However, compound **28** is limited by poor oral bioavailability with a low* F*% of 14.9.

Compound **29** (ODM-203), as a potent VEGFR2/FGFR inhibitor, exerts remarkable inhibitory activities against the VEGFR and FGFR family with IC_50_ values in the low nanomolar range. Moreover, it is selective for VEGFR and FGFR over other kinases. In vitro and in vivo pieces of evidence have proven the significant tumor suppression role (TGI = 92%) of **29** in FGFR-dependent cell lines RT4 [89]. Notably, it has been evaluated in clinical trials for solid tumor therapy (NCT02264418) [90].

In 2016, Yan et al*.* designed and synthesized dual inhibitors of FGFR and VEGFR2 through knowledge- and structure-based methods [91]. Among them, molecule **30**, containing a 3-benzimidazol-5-pyridine alkoxy-1H-indole scaffold, shows significant inhibitory activities against VEGFR2 and FGFR1-4 with IC_50_ values of 7.5 nM, 0.9 nM, 2.0 nM, 2.0 nM and 6.1 nM, respectively. Furthermore, compound **30** not only potently inhibits a panel of FGFR-amplified cell lines in vitro, but also presents considerable bioavailability (33% F) and tumor growth suppression (TGI = 96.9%) in vivo.

### Dual VEGFR2–c-Met inhibitors

**Fig. 5:**
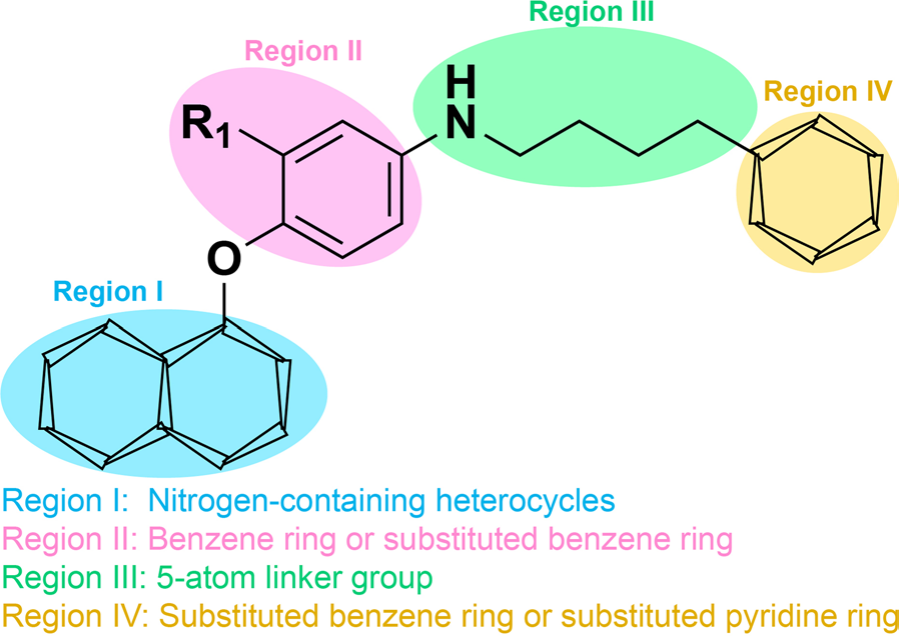
Structural formulae of dual VEGFR2/c-Met inhibitors

**Fig. 6:**
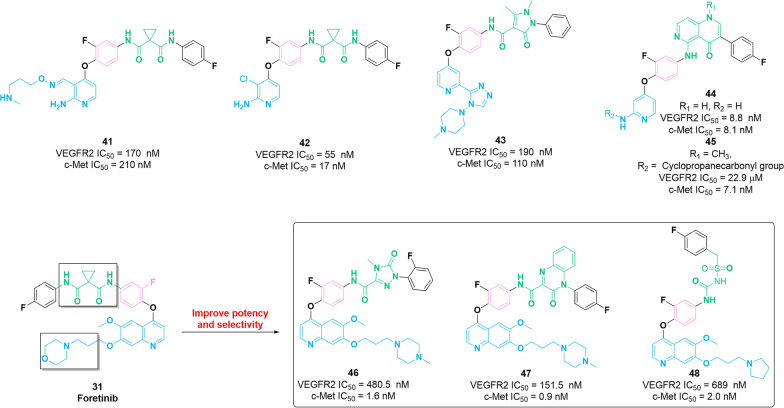
Chemical structure and properties of dual VEGFR–c-Met inhibitors **41**–**48**

**Fig. 7:**
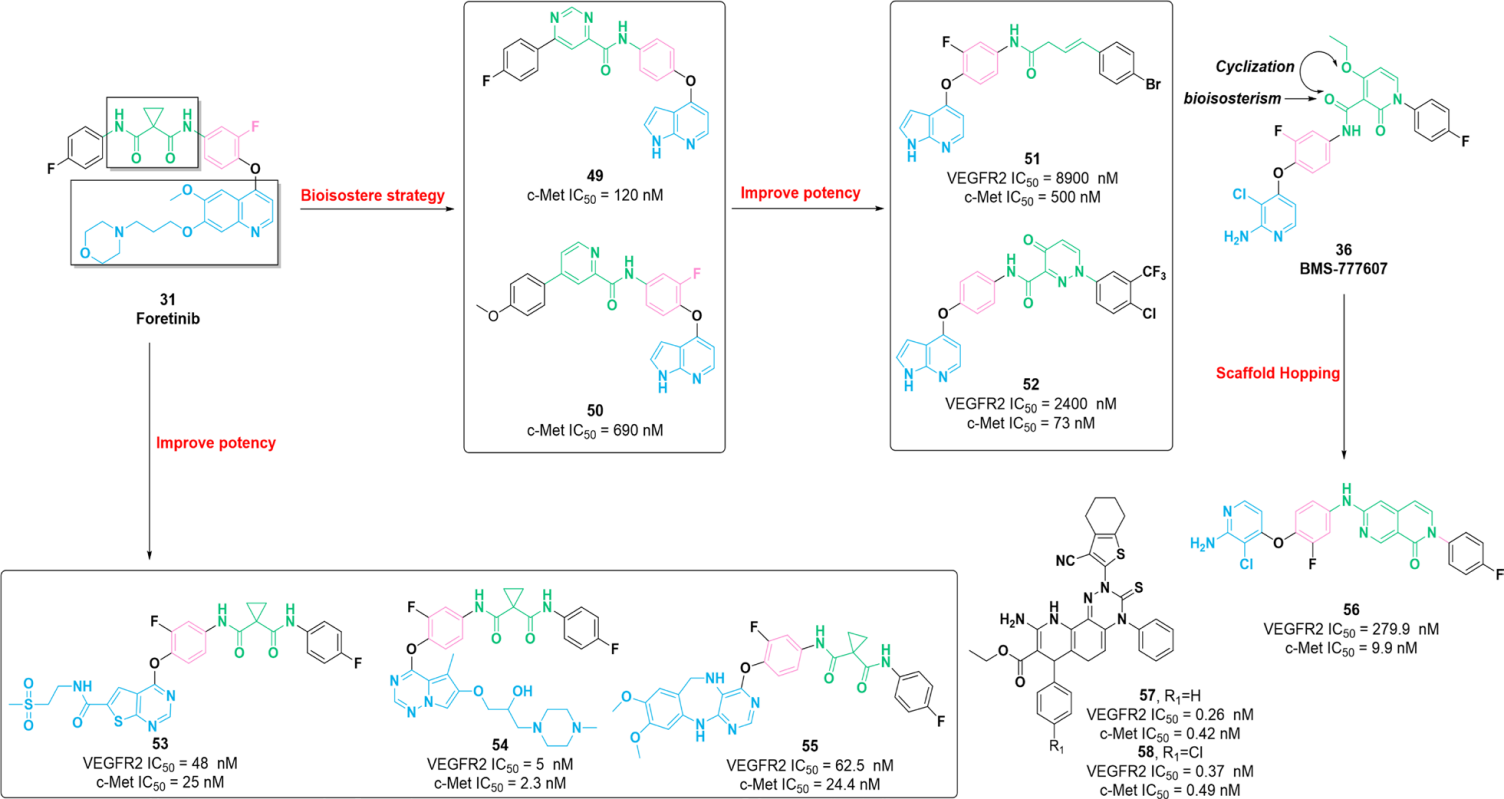
Chemical structures of dual VEGFR–c-Met inhibitors **49–58** and their inhibitory activities against VEGFR2 and c-Met

Although the combination of VEGFR inhibitors and c-Met inhibitors inhibits both VEGFR and c-Met signaling pathways, it significantly suppresses the development of different types of tumors [92]. Therefore, the identification of dual-target VEGFR/c-Met inhibitors has been boosted. As shown in Table 3, several pyridine- or pyrimidine-based inhibitors of VEGFR2/c-Met have been identified and are being used in clinical trials, including **31** (foretinib)**, 32** (golvatinib), **33** (dovitinib), **34** (tivozanib), **35** (BMS-794833), **36** (BMS-777607), **37** (MGCD-265), **38** (AC480), **39** (CP-724714) and **40** (AMG-458). These inhibitors are characterized by acting on multiple targets and exerting remarkable potency against tumor cells. Their active scaffolds warrant further investigation, thereby promoting the development of VEGFR/c-Met dual inhibitors. As shown in Fig. 5, most VEGFR2/c-Met dual inhibitors shared the following characteristics: (i) in the region I, different nitrogen-containing aromatic heterocycles, including pyridine, pyrrolidine and quinazoline, can be introduced to form a hydrogen bond with the amino acid residues of VEGFR (Cys919) and c-Met (Met1160). Additionally, the side chains of aromatic heterocycles have a significant effect on the affinity of molecule and target; (ii) region II is composed of a pyridine ring or benzene ring, which can be either unsubstituted or mono-substituted; (iii) in the region III, the introduction of a flexible chain or rigid ring structure (5-atom linker group), containing one or more hydrogen bond donors or receptors, promotes the efficiency of the molecule. Hydrogen bonds formed between this region with the amino acid residues of VEGFR (Lys868, Asp1046, etc.) and c-Met (Asp1220, Lys1110, Leu1245, etc.); (iv) region IV is made up of a six-membered aromatic heterocyclic ring, which can be either unsubstituted, mono- or di-substituted [93]. Here, we summarized the major achievements of dual VEGFR/c-Met inhibitors, and their chemical structure, potency and development are illustrated in Figs. 6 and 7.

**Table 3:** Summary of clinically approved dual VEGFR–c-Met inhibitors

Drugs	Chemical structure	Target	Tumor types	Phase	Ref.
Foretinib (31)		VEGFR2/3, c-Met, Tie-2	Gastric tumor and head/neck tumor	II	[94]
Golvatinib (32)		VEGFR2, c-Met	Head and neck tumor, liver tumor	II	[94]
Dovitinib (33)		FLT3, FGFR1/3, VEGFR1,2,3, EGFR, c-Met	Solid tumor	IV	[95]
Tivozanib (34)		VEGFR1/2/3, c-Met, PDGFR, c-Kit	Advanced renal cell carcinoma	III	[96]
BMS-794833 (35)		VEGFR2, c-Met, Ron, Axl, FLT3	Gastric tumor	I	[93]
BMS-777607 (36)		VEGFR, c-Met, Ron, Axl,	Advanced solid tumor	II	[97]
MGCD265 (37)		VEGFR1/2, c-Met, Ron	Non-small cell lung tumor	II	[98]
AC480 (38)		VEGFR2, HER1/2/4, c-Kit, Met, Lck	Advanced solid tumor	I	[99]
CP-724714 (39)		HER2, EGFR, VEGFR2, c-Met	Advanced solid tumor	II	[100]
AMG-458 (40)		VEGFR2, c-Met	Solid tumor	Non-medicinal	[101]

Pyridine/pyrimidine scaffolds have been widely applied in RTK inhibitors including VEGFR. Particularly, the pyridine motif stretches into the ATP-binding pocket of the target protein and interacts with the adjacent residues in the hinge region [102]. In 2016, a series of aminopyrimidine derivatives were designed and synthesized to evaluate their inhibitory activities against VEGFR2 and c-Met [103]. Among these compounds, molecule **41** has considerable potency against VEGFR2 and c-Met with IC_50_ values of 170 nM and 210 nM, respectively. In the following year, Zhao et al*.* identified compound **42** based on the structure of molecule **41**, which is a potent VEGFR2/c-Met dual inhibitor [104]. Moreover, **42** presents a cell active and potent potency against VEGFR2 (IC_50_ = 55 nM) and c-Met (IC_50_ = 17 nM) in vitro*.* Preliminary SAR for these compounds demonstrated that the introduction of a chlorine atom can positively regulate the kinase inhibitory activities against VEGFR2 and c-Met. In the same year, Gu et al*.* designed and synthesized a series of novel VEGFR2/c-Met dual inhibitors. Among them, compound **43** has a promising potency against targets [IC_50_ = 160 nM (VEGFR2) and 110 nM (c-Met)], and inferior antiproliferative effects on human vascular endothelial cells HUVEC and BaF3-TPR-Met cells compared with that of positive control **6** [105]. Docking studies further confirmed that molecule **43** occupies the ATP-binding pocket of VEGFR2 and c-Met, and its pyridine and triazole moiety forms hydrogen bonds with the amino acid residues of VEGFR2 (Cys919) and c-Met (Tyr1159 and Met1160). Moreover, the 5-atom linker group (pyrazolone moiety) of compound **43** generates at least one hydrogen bond with the amino acid residues of VEGFR2 (Val898 and Lys868) and c-Met (Asp1222). Similarly, using the scaffold hopping strategy, compound **44** containing the 1,6-naphthyridine scaffold is identified as a potent dual inhibitor of VEGFR2 and c-Met with IC_50_ values of 68 nM and 9.8 nM, respectively [106]. In addition, **44** exerts an unfavorable pharmacokinetic profile (***F***% = 12, CL = 5.0 L/h/kg). Further optimization has been performed based on the structure of molecule **44**, and as a result, compound **45** was identified as a potent inhibitor of c-Met (IC_50_ = 7.1 nM) with selectivity over VEGFR2. PK studies revealed moderate clearance (CL = 0.02 L/h/kg) and suitable oral bioavailability (F = 57%) of **44**. SAR studies showed that substitutions on the 2-aminopyrimidine skeleton were more resistant to c-Met efficacy than VEGFR2.

The quinoline core is widely used to design several active molecules with different biological properties. In 2016, based on the SAR studies of compound **31**, molecule **46** was identified as an effective inhibitor of c-Met (IC_50_ = 1.57 nM) by Liu et al*.* [107]. The selectivity of **46** for c-Met is 306 times higher than that of VEGFR2 and 100 times higher than that of PDGFRα and Ron. Additionally, **46** exerts significant inhibitory potency against different types of tumor cells (HT-29, H460, A549 and MKN-45) with the IC_50_ values of 80 nM, 140 nM, 110 nM and 30 nM, respectively. Further SAR studies revealed that the replacement of the 5-atom linker group (the cyclopropane-1,1-dicarboxamide moiety) of **31** with the 5-oxo-4,5-dihydro-1*H*-1,2,4-triazole-3-carboxamide fragment and structural modification (methyl, ethyl or cyclopropyl group) on the 4-position of the 1,2,4-triazole skeleton can be tolerable. Interestingly, the introduction of a fluorine atom at 2-position of benzene ring at the end would be beneficial to the potent cytotoxicity. Similarly, the 3-oxo-3,4-dihydroquinoxaline-2-carboxamide moiety displayed similar properties to the cyclopropane-1,1-dicarboxamide moiety, in particular containing hydrogen bond donor and acceptor. These studies accelerated the identification of compound **47**, which contains the 3-oxo-3,4-dihydroquinoxaline-2-carboxamide moiety at the 5-atom linker group [108]. **47** exerts superior inhibitory activities against c-Met (IC_50_ = 0.9 nM) compared with that of molecule **31** (IC_50_ = 1.41 nM). Furthermore, **47** exhibits high inhibitory potency against c-Kit (IC_50_ = 2.45 nM) and exerts considerable efficacy against Ron, VEGFR2 and FLT3 with IC_50_ values of 82.56 nM, 151.47 nM and 268.81 nM, respectively. Further in vitro studies showed that **47** displayed remarkable cytotoxicity (IC_50_ values in the nanomolar concentration range) against different types of tumor cells. Regrettably, studies in vivo are lacking. In 2019, a c-Met inhibitor **48** containing 4-phenoxyquinoline skeleton and sulfonylurea moiety is discovered by the structural optimization of compound **31** [109]. It presents an excellent inhibitory effect against c-Met with an IC_50_ value of 1.98 nM. Moreover, **48** is highly selective for c-Met over 347 times higher than that of VEGFR. **48** has strong antiproliferative activity against different types of tumor cell lines with nanomolar potency in vitro*.* The SAR studies demonstrated that the introduction of sulfonylurea fragment as the 5-atom linker group could maintain remarkable potency. In recent years, pyrrolopyridine derivatives, as biologically active molecules, occupy a unique place in medicinal chemistry [110]. Zhu et al*.* identified compounds **49** and **50** as potent inhibitor of c-Met (IC_50_ = 120 nM and 670 nM, respectively) using the bioisostere strategy, which possesses excellent cytotoxicity activities against different types of tumor cells [111, 112]. Based on the structures of **49** and **50**, compound **51**, containing an N-acylhydrazones group, was identified as a potential c-Met inhibitor (IC_50_ = 0.5 μM) by Wang et al*.* [113]*.* In addition, **51** shows a considerable selectivity profile for c-Met over other kinases (FLT3, VEGFR2 and EGFR) and exerts significant inhibitory activities against diverse types of tumor cells through arresting the cell cycle in the G2/M-phase inducing cell apoptosis. Similarly, compound **52** was identified by the same team by introducing 4-oxo-pyridazinone fragment into 5-atom linker group of molecule **49**. It displays remarkable inhibitory activities against c-Met with an IC_50_ value of 73 nM. The selectivity of **52** for c-Met is approximately 15 times higher than VEGFR2 and c-Kit and, specifically, 7 times higher than that of FLT3. In vitro assay showed that molecule **52** exerts favorable inhibitory activities against different types of tumors [114].

Similarly, based on the structure of molecule **31**, a series of thieno[2,3-*d*]pyrimidine derivatives were prepared as potent dual inhibitors of VEGFR2 and c-Met [115]. Among these compounds, molecule **53** exerts remarkable inhibitory activities against VEGFR2 and c-Met with IC_50_ values of 48 nM and 25 nM, respectively. Docking studies demonstrated that the thieno[2,3-*d*]pyrimidine scaffold of molecule **53** generates hydrogen bonds with the amino acid residues of VEGFR2 (Cys919) and c-Met (Met1160). Additionally, hydrogen bonds formed between 4-fluoro-phenyl-cyclopropane-1,1-dicarboxamide fragment with the amino acid residues of VEGFR (Asp1046 and Lys868) and c-Met (Phe1223). In 2018, compound **54** containing pyrrolo[1,2-*f*][1,2,4]triazine core is identified as a remarkable dual inhibitor of VEGFR2 (IC_50_ = 5.0 nM) and c-Met (IC_50_ = 2.3 nM) [116]. **54** showed remarkable inhibitory activities against BaF3-TPR-Met, HUVEC and different types of tumor cells. Besides, compound **54** possesses favorable physiochemical properties and an excellent pharmacokinetic profile (***F***% = 98.1). Further docking studies revealed that molecule **54** can completely occupy the ATP-binding pocket of c-Met and VEGFR2, thereby generating important ligand interactions. In the same year, Huang et al*.* identified compound **55** as a c-Met inhibitor, which bears 6,11-dihydro-5*H*-benzo[e]pyrimido[5,4-*b*] [1, 4] diazepine scaffold [117]. Enzymatic inhibition assay showed that **55** exerts notable inhibitory potency against c-Met and VEGFR2 with IC_50_ values of 24.4 nM and 62.5 nM, respectively. Compound **55** has a selectivity profile for VEGFR2 and c-Met over other kinases. Furthermore, molecule **55** possesses favorable in vitro potency and moderate oral bioavailability (***F***% = 39). Further in vivo pieces of evidence demonstrated that compound **55** had a considerable therapeutic effect (TGI = 64.5%).

In 2019, Zhuo et al*.* designed and synthesized a series of 2,7-naphthyridinone-based c-Met inhibitors through knowledge- and structure-based methods [118]. In vitro assay showed that molecule **56**, as the most promising inhibitor, possesses a potent potency for c-Met (IC_50_ = 9.9 nM), and the selectiveness for c-Met is 28 times higher over VEGFR2. Additionally, compound **56** possesses favorable pharmacokinetic profile (***F***% = 54) and excellent in vivo efficacy (TGI = 95%) in mouse xenograft tumor models. Based on these studies, 2,7-naphthyridinone may be a promising skeleton for future drug development. In 2020, compounds **57** and **58** containing tetrahydrobenzo[*b*]thiophene scaffold are identified as multi-target RTK inhibitors [119]. Specifically, **57** and **58** possess remarkable potency against multi-kinase, including c-Met and VGEFR2, with IC_50_ values in the low nanomolar to picomolar concentration range. Moreover, they also exert antiproliferative activities in vitro against the six typical tumor cells, including A549, H460, HT-29, MKN-45, U87MG and SMMC-7721. However, in vivo studies are still lacking.

### Dual VEGFR2–BRAF inhibitors

**Fig. 8:**
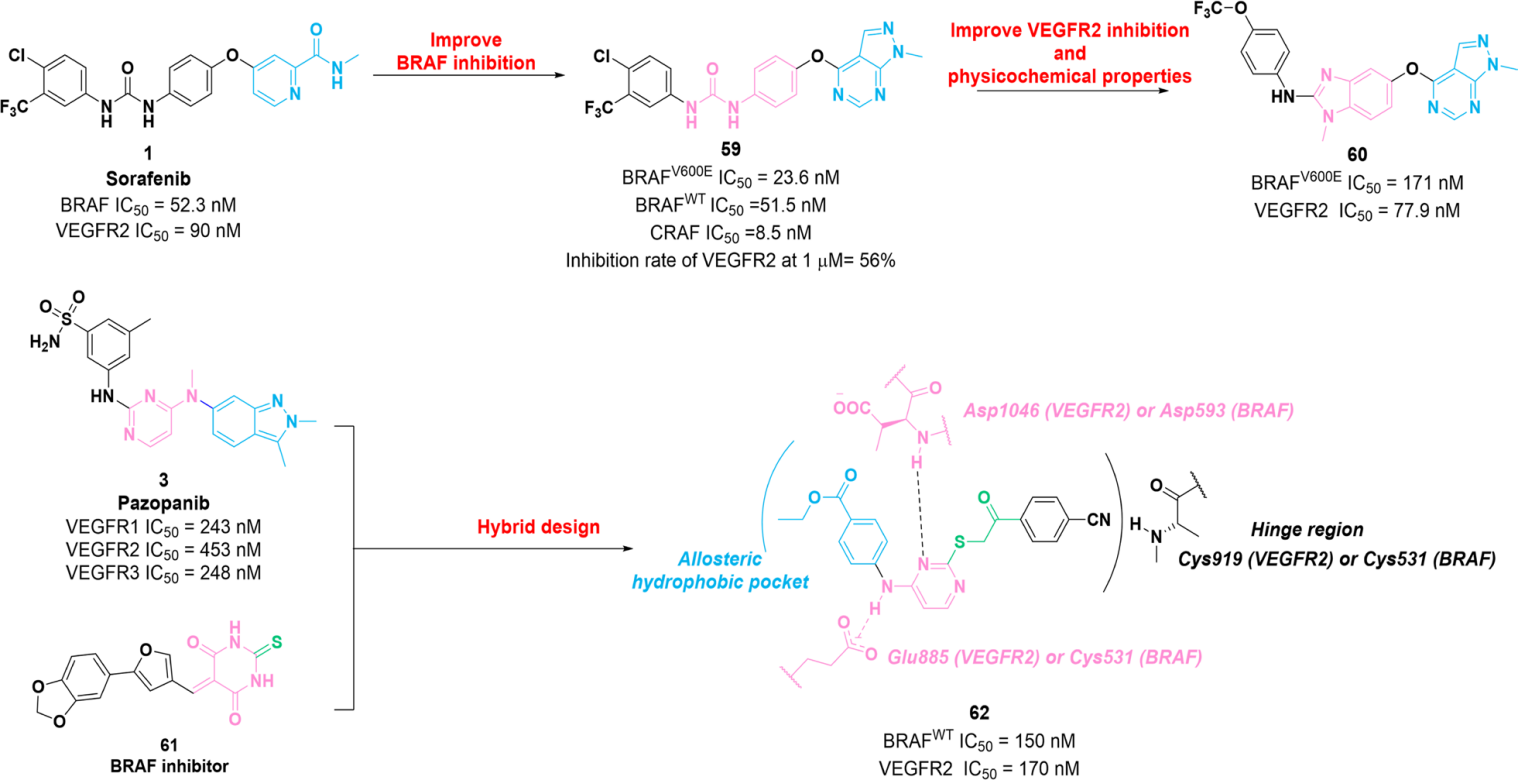
Chemical structures of dual VEGFR–BRAF inhibitors **59**, **60** and **62** and their inhibitory activities against VEGFR2 and BRAF

As we mentioned earlier, combination therapy involving VEGFR inhibitors and BRAF inhibitors has been identified as an effective therapeutic strategy [120]. Notably, RAF-265, a VEGFR2–BRAF dual inhibitor, has demonstrated its efficacy and safety profile in a I/II clinical phase trial (NCT00304525). Currently, several dual VEGFR2–BRAF inhibitors have been identified to suppress tumor growth. Their chemical structure, in vitro and in vivo potencies, and optimizations are illustrated in Fig. 8.

In 2017, Fu et al*.* identified compound **59** by using a structure-based drug design as an encouraging RAF inhibitor [121]. It presents remarkable inhibitory potency against c-RAF, wild-type BRAF and BRAF^V600E^ with IC_50_ values of 8.5 nM, 51.5 nM and 23.6 nM, respectively. Additionally, the strong antiproliferative activity of **59** against four types of tumor cell lines with micromolar potency in vitro, and superior selectivity for RAF compared to other kinases have been confirmed. Particularly, **59** presents moderate inhibitory activity against VEGFR2 kinase. (The inhibition rate of VEGFR2 at 1 μM was 56%.) Mechanistic studies revealed that **59** exerts antiproliferative activities on A375 and HT-29 cells by arresting the cell cycle progression in G0/G1 stage and significantly suppressing RAS/RAF/MEK/MAPK signaling pathways. The following year, compound **60** was developed based on the structural optimization of **59**, which has excellent selectivity for VEGFR2 (IC_50_ = 77.9 nM) and BRAF^V600E^ (IC_50_ = 171 nM) over wild-type BRAF and other protein kinases [122]. Docking studies and molecular dynamics simulations demonstrated that **60** adopts a similar binding mode to that of compound **1** at the ATP-binding sites of BRAF^V600E^ and VEGFR2. These studies indicated that **60** can sever as a lead compound for the identification of potent BRAF^V600E^/VEGFR2 dual inhibitors.

In 2019, compound **62** was identified as an effective wild-type BRAF/VEGFR2 dual inhibitor through structural hybridization between VEGFR inhibitor **3** and BRAF inhibitor **61** [123]. **62** exerts potent inhibitory activities against wild-type BRAF and VEGFR2 with IC_50_ values of 150 nM and 170 nM, respectively. Moreover, **62** possesses antiproliferative activity against MCF-7 and T-47D cells with IC_50_ values in the micromolar concentration range. Docking studies revealed that **62** can bind to the active sites of VEGFR2 and BRAF, thereby accomplishing the key binding interactions.

### Dual VEGFR2–HDAC inhibitors

**Fig. 9:**
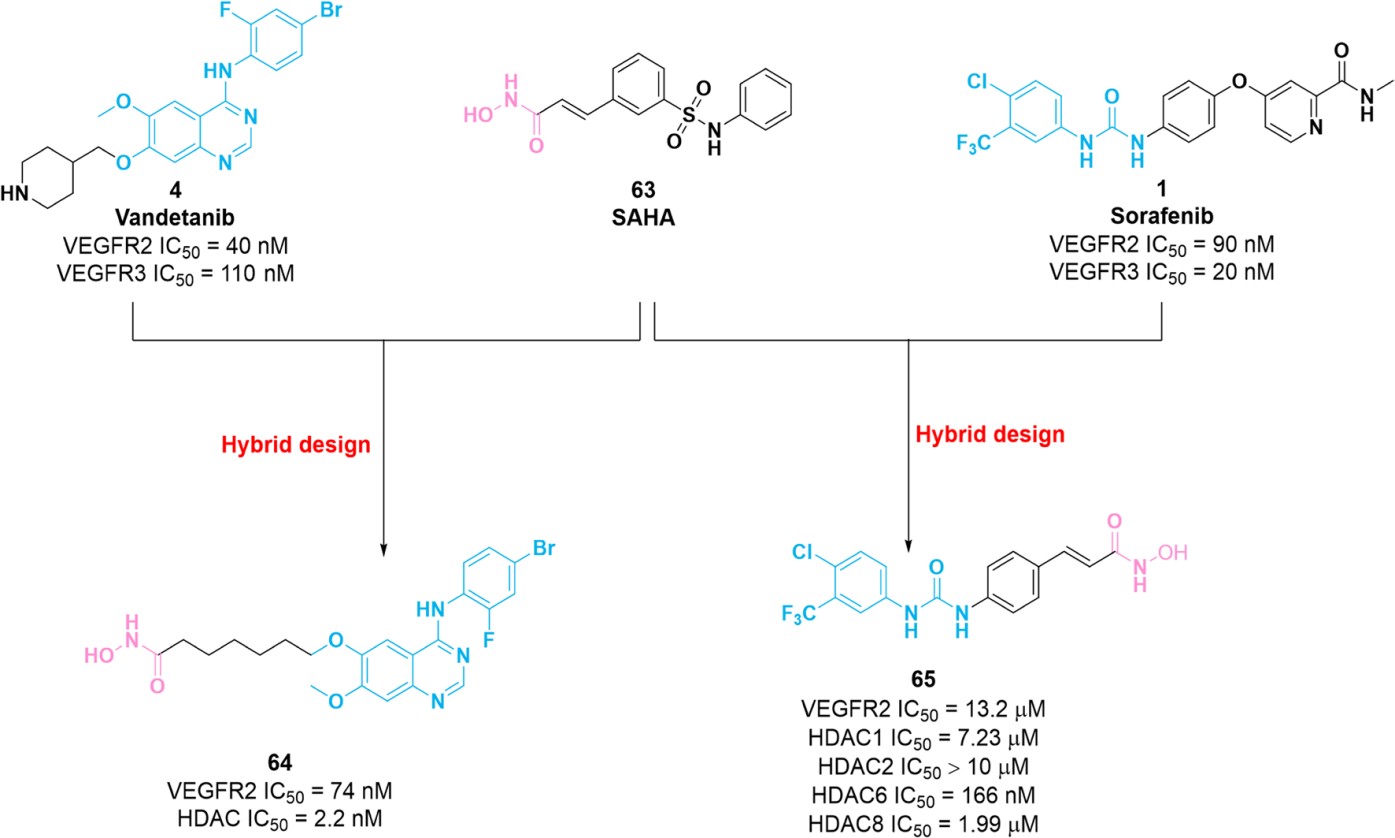
Chemical structures of dual VEGFR–HDAC inhibitors **64** and **65** and their inhibitory activities against VEGFR2 and HDAC

Recent studies have shown that the combination of VEGFR inhibitors and HDAC inhibitors exerts promising potency in vitro and in vivo*.* Structurally, HDAC inhibitors generally consist of three parts: a zinc-binding group (ZBG), an appropriate linker and a capping group (CAP group). Notably, SAR studies showed that modification of the CAP group of HDAC inhibitor is tolerable. Hence, the CAP group could hybridize with VEGFR2 inhibitors for the identification of dual-target inhibitors [124]. In 2016, a series of VEGFR2/HDAC dual inhibitors containing *N*-phenylquinazolin-4-amine and hydroxamic acid moieties were obtained based on the parent compounds **4** and **63** (SAHA) [125]. Among them, molecule **64** (Fig. 9) possesses remarkable potency against VEGFR2 (IC_50_ = 74 nM) and HDAC (IC_50_ = 2.2 nM) and shows favorable inhibitory activities against human breast tumor cells MCF-7 with an IC_50_ value of 850 nM. Unfortunately, the selectivity profile of compound **64** for HDAC family members is lacking. Recently, by incorporating pharmacophores of VEGFR inhibitor **1** and HDAC inhibitor **63**, a series of phenylurea hydroxamic acids were synthesized to evaluate their inhibitory activities against VEGFR2 and HDAC [126]. Among these compounds, molecule **65** (Fig. 9) potently inhibits HDAC6 (IC_50_ = 166 nM) and is slightly selective for HDAC6 over other HDAC1, HDAC2 and HDAC8. Furthermore, **65** exerts weak potency against VEGFR2 with an IC_50_ value of 13.2 μM. The co-crystal structure of **1** in complex with VEGFR2 (PDB: 3EWH) showed that the key *N*-methyl-2-pyridinecarboxamide group is of significance in the potency against VEGFR2, which is inserted into the hinge region through generating two hydrogen bonds with Cys919 [127]. Thus, unreasonable modification of this region of molecule **1** can lead to a loss of inhibitory activity.

**Fig. 10:**
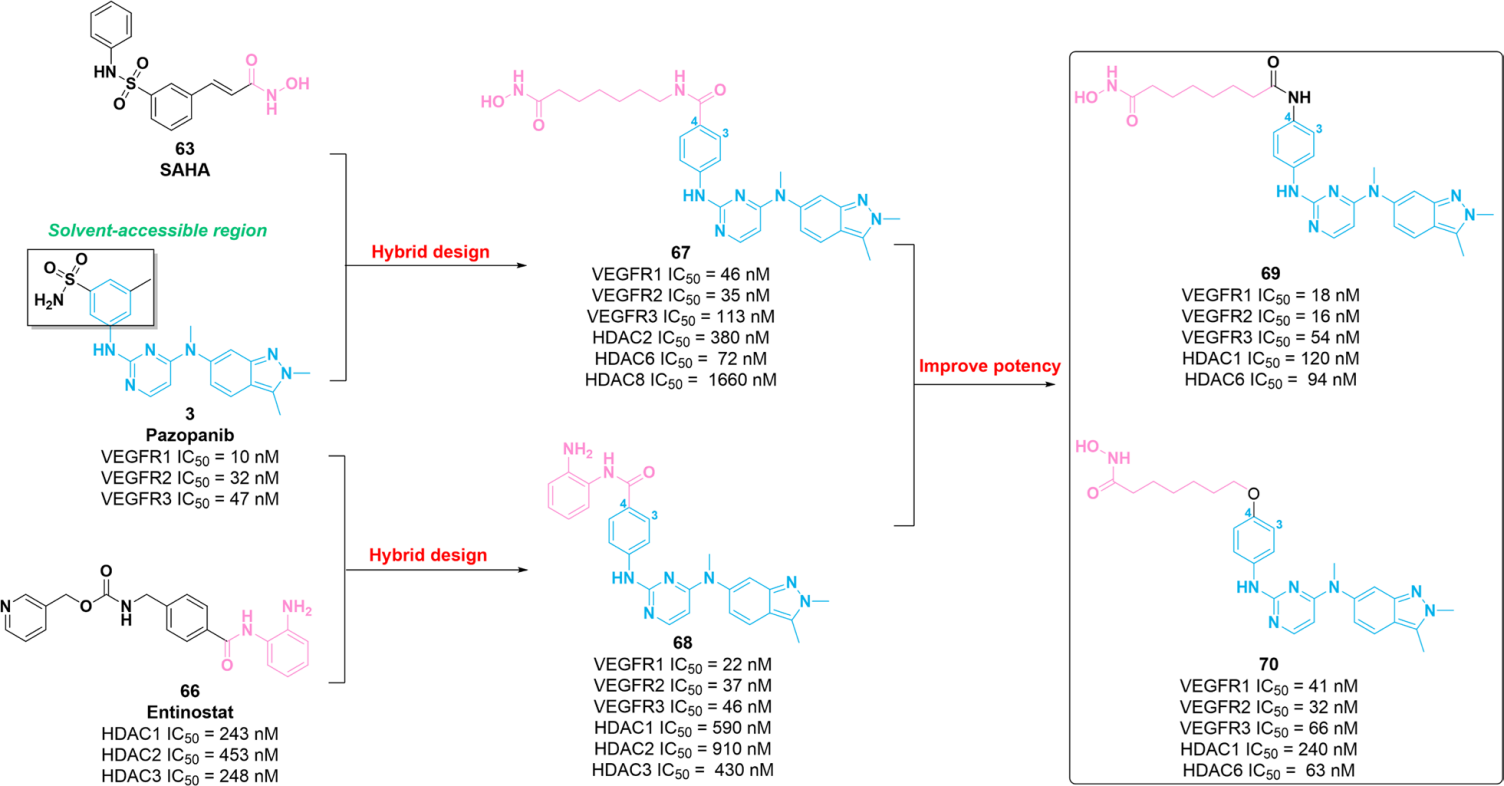
Chemical structures of dual VEGFR–HDAC inhibitors **67–70** and their inhibitory activities against VEGFR2 and HDAC

**Fig. 11:**
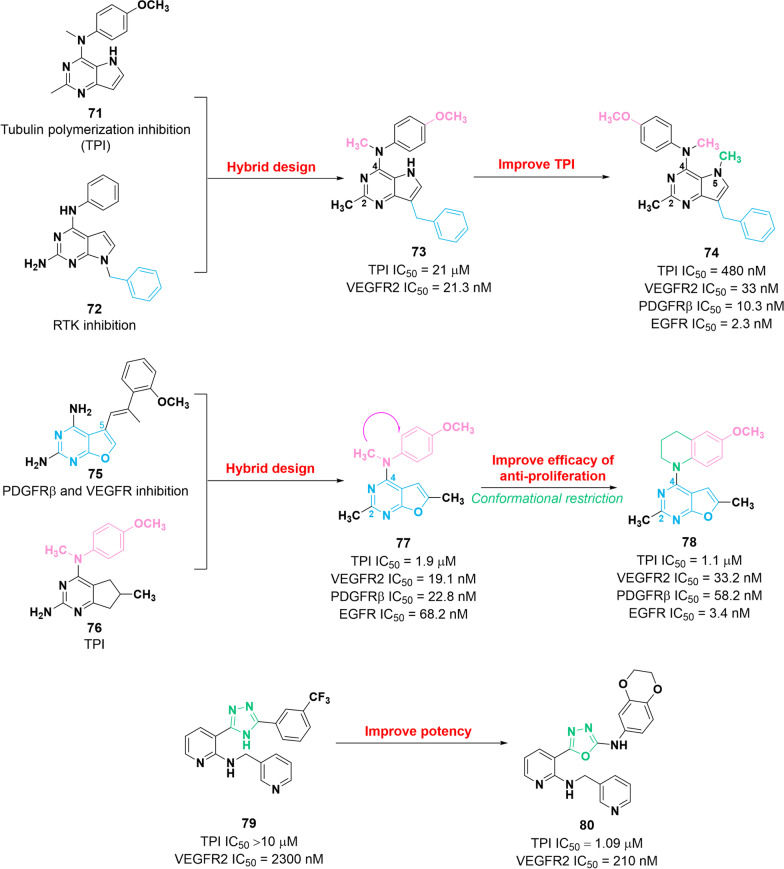
Chemical structures of dual VEGFR–tubulin inhibitors **73**, **74**, **77**, **78**, **79** and **80** and their inhibitory activities against VEGFR2 and tubulin polymerization (TPI)

In 2018, Zhang et al. generated two series of dual VEGFR–HDAC inhibitors using the pharmacophore of VEGFR inhibitor **3** as the CAP group and the diverse linker group, hydroxamic acid or *ortho*-aminoanilide as the ZBG [128]. Of these, compounds **67** and **68** (Fig. 10) exert potent potency against VEGFR and HDAC with IC_50_ values in the nanomolar or low micromolar concentration range. Specifically, compound **67** exerts considerable HDAC2/6 inhibitory potency and superior HDAC8 inhibition compared with that of molecule **63**. Molecule **68** also possesses comparable efficacy against HDAC1/2/3 compared with that of molecule **66** (Entinostat). Additionally, compared with compound **3** (VEGFR2 IC_50_ = 32 nM), **67** and **68** show similar inhibitory activities against VEGFR2 with IC_50_ values of 35 nM and 37 nM, respectively. Other kinases (VEGFR1, VEGFR3, PDGFRβ, FGFR, C-Fms and c-Kit), which are tumor-related targets inhibited by **3**, could be potently inhibited by molecules **67** and **68**. SAR of **67** and **68** can be briefly summarized as follows: (i) structural modification of the solvent-exposed phenyl moiety of molecule **3** was well tolerated for its kinase inhibition profile; and (ii) substitution in position 4 of the solvent-exposed phenyl group is favorable for the potency against HDAC and VEGFR2. In addition, compound **68** exerts desirable pharmacokinetic profiles (***F***% = 72) and moderate in vivo antitumor effects (TGI = 40%) in a HT-29 xenograft model. In 2022, compounds **69** and **70** (Fig. 11) were developed based on structure optimization of **67**, which have promising potency for HDACs and stronger inhibitory activities against VEGFRs compared with that of molecule **67** [129]. Furthermore, molecules **69** and **70** showed favorable antiproliferative activities against different types of tumor cells with IC_50_ values in the micromolar concentration range.

### Dual VEGFR2–tubulin inhibitors

The effect of combination therapy with tubulin inhibitors and VEGFR2 inhibitors has been confirmed by several studies [130, 131]. Presently, several dual VEGFR2–tubulin inhibitors have been identified to exert potent antitumor activities. Their chemical structure, in vitro potency and optimization are illustrated in Fig. 11.

In 2014, a series of VEGFR2–tubulin inhibitors were prepared by introducing the 7-benzyl moiety of RTK inhibitor **72** into the core of tubulin inhibitor **71** [132]. Among them, molecule **73** exerts significant inhibitory efficacy against VEGFR2 and tubulin polymerization with IC_50_ values of 21.3 nM and 21 μM, respectively. Preliminary SAR studies showed that (i) 7-benzyl fragment plays a key role in the maintenance of potency against VEGFR2; and (ii) N–CH_3_ and O–CH_3_ are essential for the inhibitory activity against VEGFR2 and microtubule. In vitro assays showed that **73** possesses potent antiangiogenic and antiproliferative activity. Specifically, **73** presents remarkable inhibitory activities against βIII-tubulin-overexpressing HeLa cells (IC_50_ = 280 nM) and P-gp-overexpressing ADR-RES cells (IC_50_ = 700 nM), thereby theoretically reversing βIII-tubulin- and P-gp-overexpression-induced drug resistance. Additionally, **73** shows potent antitumor and antimetastasis effects in in vivo tumor models. In 2017, compound **74** was developed based on the structure of **73**, which presents a potent potency against VEGFR2 (IC_50_ = 33 nM), tubulin polymerization (IC_50_ = 480 nM), EGFR (IC_50_ = 2.3 nM) and PDGFRβ (IC_50_ = 10.3 nM) in vitro [133]*.* Furthermore, **74** presents superior cytotoxicity activities against βIII-tubulin-overexpressing (IC_50_ = 250 nM) and P-gp-overexpressing (IC_50_ = 70 nM) tumor cells compared with that of **73**. Further SAR studies demonstrated that the N4-CH_3_ and N5-CH_3_ groups play a key role in the inhibitory potency against tubulin polymerization. Structurally, the N5-CH_3_ group is thought to favor the formation of conformational rigidity, thereby improving efficacy. Moreover, the 2-CH_3_ group was substituted with a 2-amino moiety, leading to decreased inhibitory activity against tubulin polymerization.

In another report, hybridization of the pharmacophores of RTK inhibitor **75** and tubulin inhibitor **76** in a single molecule facilitated the discovery of compound **77** [134]. This molecule exerts favorable potency against EGFR, VEGFR2, PDGFRβ, and tubulin polymerization with IC_50_ values of 68.2 nM, 19.1 nM, 22.8 nM and 1900 nM, respectively. Furthermore, in vitro and in vivo pieces of evidence proved the superior potency of compound **77** on proliferation inhibition and repression of tumor angiogenesis compared with that of docetaxel. Based on the structure of compound **77** and further ligand design, compound **78** was further discovered to be more potent than compound **77** for tubulin polymerization (IC_50_ = 1100 nM) and EGFR (IC_50_ = 3.4 nM) [135]. However, **78** exerts inferior potency against VEGFR2 (IC_50_ = 33.2 nM) and PDGFRβ (IC_50_ = 58.2 nM). SAR studies demonstrated that the introduction of tetrahydroquinoline ring fragment is beneficial to improve EGFR inhibition. Moreover, **78** significantly inhibits the growth of drug resistance HeLa and SK-OV-3 cells with IC_50_ values of 9.1 nM and 19.4 nM, respectively.

Compound **80** containing 1,3,4-oxadiazole fragment is developed based on the structure optimization of weak VEGFR2 inhibitor **79**, which has a promising potency for tubulin polymerization (TPI IC_50_> 10 μM) and VEGFR2 (IC_50_ = 2300 nM) compared with that of molecule **80** (TPI IC_50_= 1090 nM, VEGFR2 IC_50_ = 210 nM) [136]. Additionally, **80** can block cell cycle progression in the G2/M-phase. Acute and repeat dose oral toxicity studies demonstrated that **80** has a favorable safety profile.

### Dual VEGFR2–ERα inhibitors

**Fig. 12:**
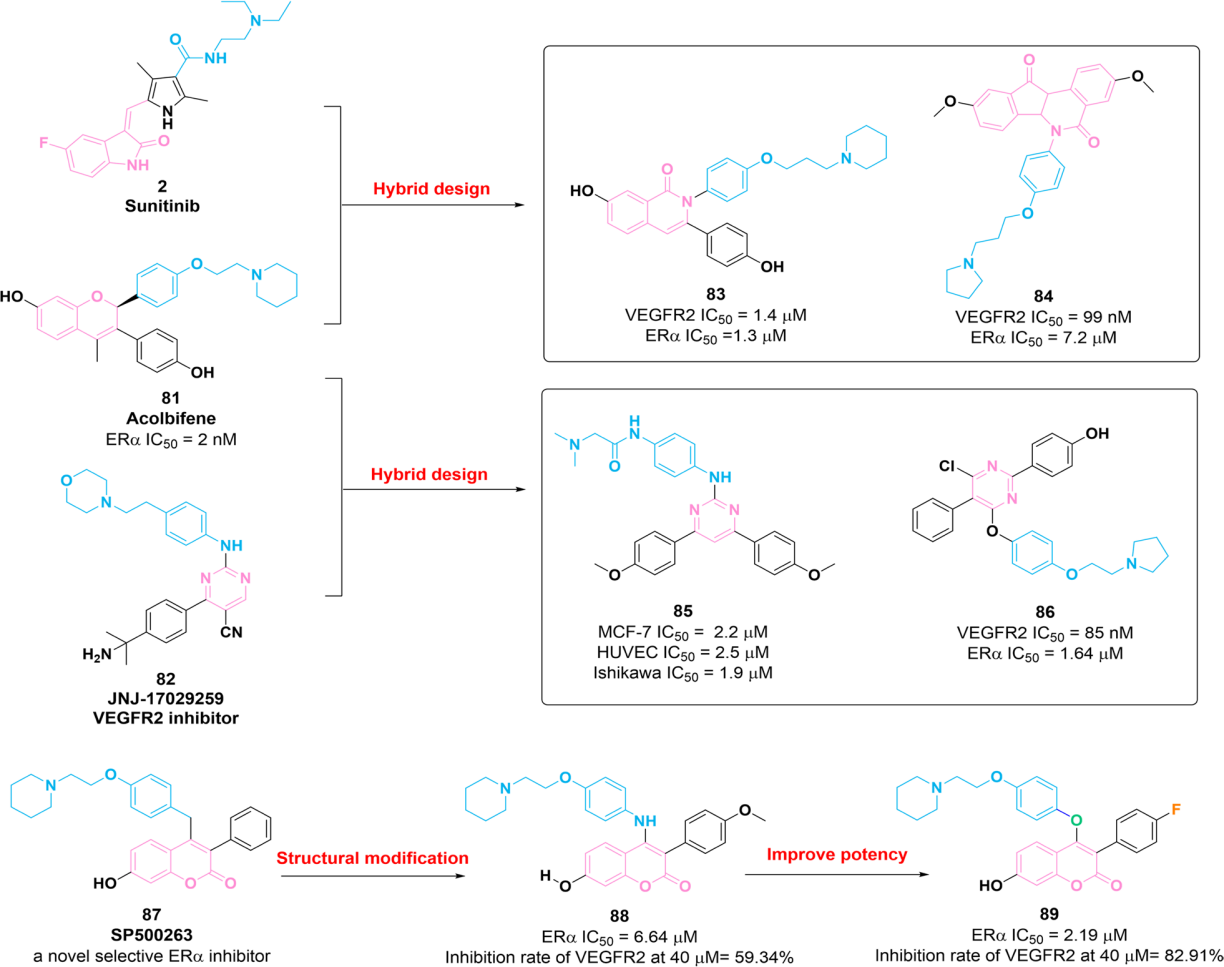
Chemical structures of dual VEGFR–ERα inhibitors **83–86**, **88** and **89** and their inhibitory activities against VEGFR2 and ERα

Combination therapy with SERMs and VEGFR inhibitors has been identified as an effective therapeutic strategy to retard SERM resistance tumor growth [137]. A number of dual *VEGFR2*–ERα inhibitors with significant anti-breast tumor activities were obtained. Their chemical structures, in vitro potency and optimization are illustrated in Fig. 12.

In 2014, based on the structures of VEGFR inhibitor **2** and SERM **81** (acolbifene), compound **83** is further discovered to present considerable potency against ERα (IC_50_ = 1.3 μM) and VEGFR2 (IC_50_ = 1.4 μM) [138]. Biological studies revealed that **83** exerts antiproliferative activity against MCF-7 cells with an IC_50_ value of 2.73 μM and possesses potential antiangiogenesis efficacy in vivo. In 2016, a series of VEGFR2/ERα inhibitors containing aryl-indenoisoquinolone core were prepared based on the structures of **2** and **81** [139]. The analogue **84** was obtained, showing a significant potency for VEGFR2 and ERα with IC_50_ values of 99 nM and 7.2 μM, respectively. Moreover, **84** shows favorable cytotoxicity activities against MCF-7, MDA-MB-231, Ishikawa and HUVEC cell lines with IC_50_ values of 1.2 μM, 0.5 μM, 8.2 μM and 800 nM, respectively. Further in vitro studies demonstrated that **84** inhibits the growth of MDA-MB-231 cells through negatively regulating VEGFR2 and the signaling transduction of the RAF-1/MAPK/ERK pathway.

In 2017, through hybridization of bioactive pharmacophores of **81** and **82**, compound **85** containing 4,6-diaryl-2-pyrimidinamine scaffold was reported as a potential agent for breast tumor therapy. It exerts favorable inhibitory activities against MCF-7, HUVEC and Ishikawa cells [140]. Chick chorioallantoic membrane (CAM) assay showed that **85** exerts significant antiangiogenesis activity in vivo. Further optimization has been performed based on the structure of molecule **85**, and as a result, compound **86** is identified as a potent inhibitor of VEGFR2 (IC_50_ = 85 nM) and ERα (IC_50_ = 1.64 μM) [141]. Furthermore, **86** possesses remarkable antiestrogenic property through downregulating the expression of progesterone receptor (PgR) mRNA in MCF-7 cells and exerts significant antiangiogenesis efficacy in vitro and in vivo.

Compound **87** (SP500263), a coumarin-based SERM, exerts a high affinity for ERα and significantly inhibits the growth of estrogen-dependent MCF-7 cells [142]. In 2017, compound **88** was developed based on the structural optimization of **87**, with potential potency against ERα (IC_50_ = 6.64 μM) and weak inhibitory activity against VEGFR2 [143]. To improve the potency, molecule **89** with favorable potency against ERα (IC_50_ = 2.19 μM) has been identified [143]. SAR studies showed that the introduction of bioisosteric O atom at 4-position of coumarin core is essential for enhancing the ERα inhibition. Compared with molecule **88**, **89** exerts superior inhibitory activities against MCF-7 and Ishikawa cells. In MCF-7 cells, **89** induces cell apoptosis and a prolonged G0/G1-phase and inhibits proliferation and migration through negatively regulating the expression of VEGFR2 and the signaling transduction of RAF-1/MAPK/ERK pathway. Collectively, the structure of the VEGFR2/ERα inhibitor is characterized by the presence of an aromatic scaffold and flexible side chain with a tertiary amine substituent at the end. The introduction of the above two pharmacophores is beneficial to the inhibitory activities against ERα and VEGFR.

### Dual VEGFR2–PIM1 inhibitors

**Fig. 13:**
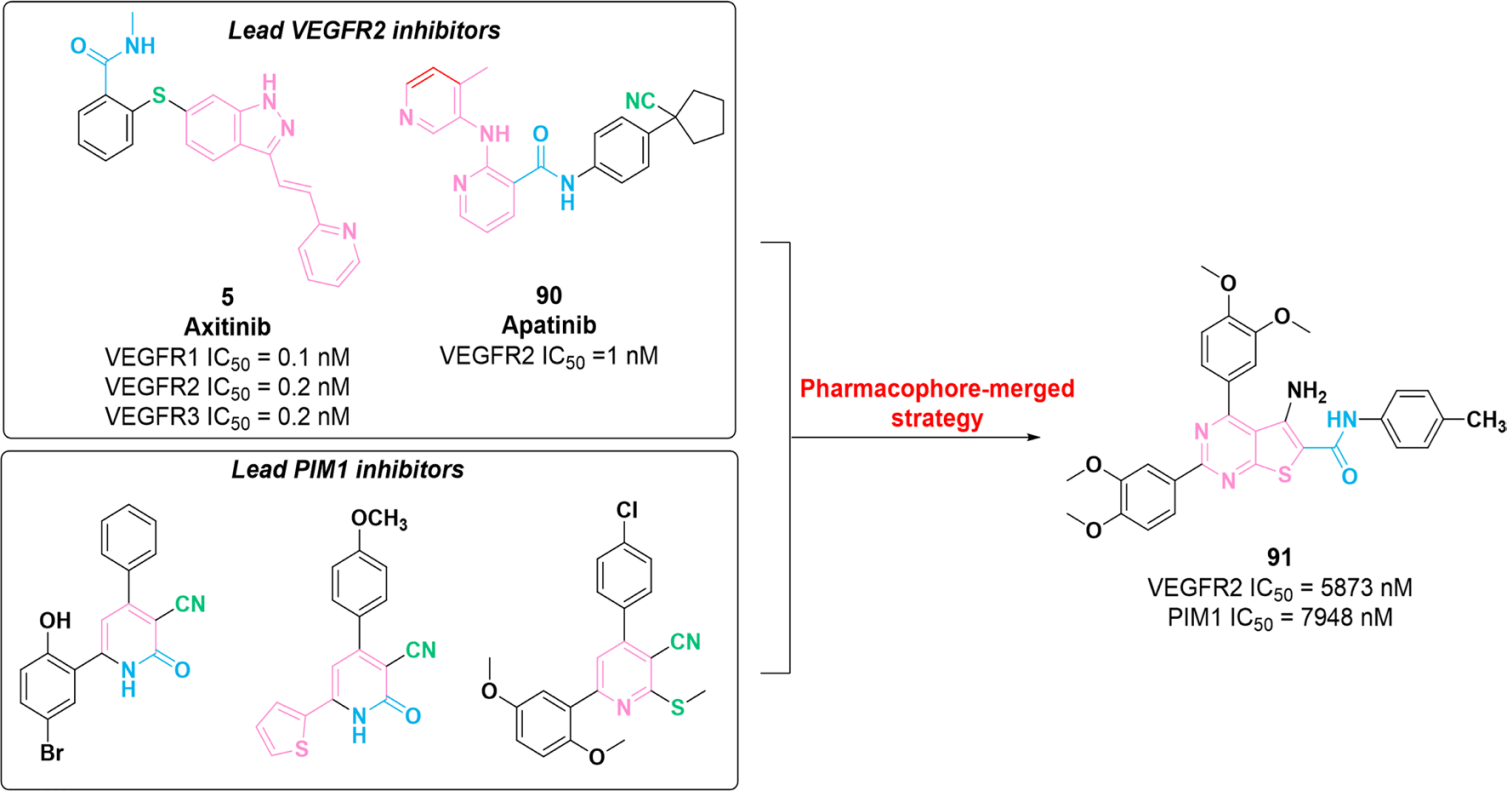
Chemical structure of dual VEGFR–PIM1 inhibitor **91** and its inhibitory activities against VEGFR2 and PIM1

The expression of PIM-1 kinase has been noted as a new resistance mechanism to VEGFR inhibitors [144]. Thus, a combination therapy involving PIM1 kinase and VEGFR inhibitor has been identified as an effective therapeutic strategy to sensitize tumor cells. In 2019, a series of PIM1/VEGFR2 dual inhibitors containing thieno[2,3-*b*]pyridine core were prepared via molecular hybridization between VEGFR inhibitors (compounds **5** and **90**) and PIM1 inhibitors (Fig. 13) [145]. Among these compounds, **91** was found to show the most potent inhibitory activities against PIM1 and VEGFR2 with IC_50_ values of 5873 nM and 7948 nM, respectively. In vitro assays showed that **91** exerted inhibitory potency against different types of tumor cells (HepG-2, Caco-2, MCF-7 and PC-3) with IC_50_ values in the nanomolar concentration range. Furthermore, **91** can positively regulate the expression of caspase 3/7 and induce apoptosis in tumor cells. Real-time PCR analysis demonstrated that **91** presents superior therapeutic potential in regulating the expression of VEGF, p53 and cyclin D compared to doxorubicin (Fig. 13).

### Dual inhibitors of VEGFR2 and other antitumor targets

Currently, several dual inhibitors of VEGFR2 and other antitumor targets were identified through serendipity or using typical design strategies, and they exerted superior potency to corresponding single-target molecules. These dual inhibitors are frequently utilized as tool compounds for investigating the synergetic interactions of VEGFR2 and other antitumor targets. In addition, they can be used as potential novel leads to discover novel dual-target antitumor agents.

**Fig. 14:**
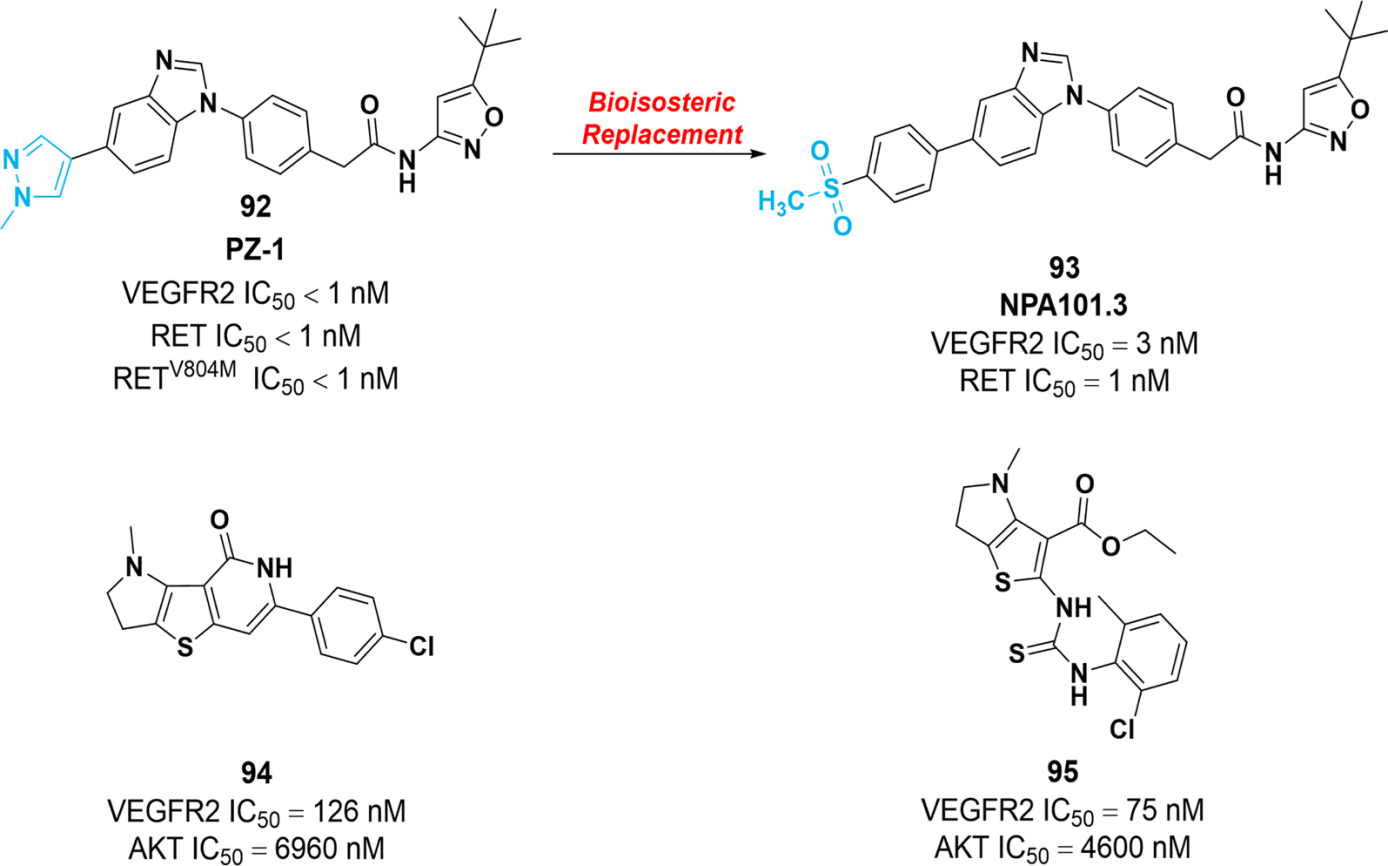
Chemical structure of dual VEGFR–RET inhibitors **92**, **93,** and VEGFR–AKT inhibitor **94** as well as their inhibitory activities against their target proteins

Under normal physiological conditions, rearranged during transfection (RET) plays an important role in the development of the kidney and nervous system. Under pathological conditions, RET rearrangements lead to the generation of chimeric genes. Mechanistically, these genes are formed by the fusion of the RET tyrosine kinase domain with the N-terminal region of other genes. Structurally, VEGFR2 and RET share a high similarity regarding their ATP-binding site. Therefore, several multi-kinase inhibitors targeting the VEGFRs, RET and other kinases are widely used in clinical practice, including **1**, **4**, **6** and **7**. In order to enhance the selectivity and reduce side effects, Brendan et al*.* discovered a dual pan-RET/VEGFR2 kinase inhibitor **92** (Pz-1) through the fragment-based chemical screen, which possessed remarkable inhibition activity with nanomolar potency against RET, VEGFR2 and RET^V804M^. Notably, in vivo results confirmed the favorable safety profile and the significant tumor growth inhibition role of **92** in nude mice implanted with RET- or RAS-transformed NIH3T3 fibroblasts [146]. In 2020, to further enhance the metabolic stability of **92**, compound **93** (NPA101.3) was identified by applying bioisosteric substitution of the molecule **92** site susceptible to demethylation (Fig. 14). Enzymatic inhibition assay showed that **93** possessed a notable inhibitory potency against both RET and VEGFR2 with IC_50_ values of 1 nM and 3 nM, respectively. Furthermore, in vitro study revealed that **93** could suppress the phosphorylation of RET oncoproteins and VEGFR2. It also remarkably inhibits the proliferation of RET-transformed Ba/F3 cells with IC_50_ values in the low nanomolar concentration range. In vivo pieces of evidence proved that compound **93** completely prevented the formation of tumors induced by RET^C634Y^-transformed cells [147].

VEGF binding to the VEGFR can lead to AKT activation, improving the proliferation, migration and invasion capacity of tumor cells. Additionally, a few studies have suggested that the resistance to VEGFR inhibitors is contributed to acquired mutations in AKT. Therefore, dual inhibition of VEGFR2 and AKT may trigger apoptosis at different focal points. In 2022, a series of VEGFR–AKT dual inhibitors containing thienopyrrole or pyrrolothienopyrimidine scaffold is prepared by Abdelnaby et al. Among them, compounds **94** and **95** showed better inhibitory activities against AKT (IC_50_ = 6.96 μM and 4.60 μM, respectively) and VEGFR2 (IC_50_ = 126 nM and 75 nM, respectively) (Fig. 14). In HepG2 cells, **94** and **95** could aggravate apoptosis by inhibiting cell proliferation and arresting cell growth in the S-phase, resulting in cell apoptosis [148].

## Conclusion and future direction

Currently, antiangiogenesis therapy based on inhibition of VEGFR is considered to be an effective clinical strategy for the treatment of solid tumors. Although VEGFR inhibitors showed prospective efficacy in clinical application, there are still barriers and challenges to surmount, such as the moderate clinical efficacy, mechanism-related toxicities and the occurrence of clinical resistance. Encouragingly, great advances have been made in identifying novel combination treatment strategies due to the progressed technologies in structural biology and pharmacochemistry. Multi-target, especially dual-target drug design, is one of the hottest areas in tumor treatment. Compared with combination chemotherapy, multi-target drugs have the advantages of synergistic antitumor effect and improved pharmacokinetic properties. Given the critical role of VEGFR in the development of tumor angiogenesis, dual-target drug design for VEGFR has become a hot topic in the drug research and development field. Several studies have proven the favorable efficacy and safety of VEGFR inhibitors and inhibitors of other tumor-associated targets (including EGFR, FGFR, BRAF, c-Met, HDAC, tubulin, ERα and PIM1) combination therapy in patients with tumors.

In general, the hybrid design strategy integrates the active group of a VEGFR inhibitor with the pharmacophore of another inhibitor of tumor-associated targets into one molecule to identify novel and potent agents. In this review, we summarize VEGFR-based dual-target inhibitors, which provide a rationale for the future design of dual-target inhibitors involving VEGFR. Clinical practice and research have demonstrated that VEGFR inhibitors have synergistic effects with various inhibitors of other tumor-associated targets [149]. However, the dual-target drug design approach has not yet been extensively applied for several targets, such as poly ADP-ribose polymerase (PARP), which possesses synergistic effects with VEGFR inhibitors [150]. Notably, several clinical studies have confirmed the efficacy and safety of VEGFR-based dual-target drugs (such as compounds **23** and **26**) for the treatment of different types of tumors. The above studies confirmed the feasibility of the VEGFR-based dual-target drug design strategy.

Yet where there are opportunities there are challenges. Firstly, identifying rational target combinations based on the correlation between reported targets and tumors is a major challenge in identifying dual-target VEGFR inhibitors. Nowadays, this is typically realized through clinical investigations and phenotype-based screening for combination treatment. Moreover, the clinical success of dual-target VEGFR inhibitors depends on the optimization of efficacy, pharmacokinetic properties and toxicity. To meet these demands, obtaining highly potent dual-target lead compound with excellent pharmacokinetic properties can serve as a starting point. A better procedure is to maximize the overlap of pharmacophores of maternal molecules to generate smaller molecules with desirable functionalities that have competent chemical space for structural optimization. Specifically, maintaining low lipophilicity and avoiding superfluous structural enlargement are the main issues to consider when optimizing the pharmacokinetic properties of dual-target VEGFR inhibitors. The pharmacophores of active parent molecules share a high degree of structural similarity. However, the dual-target molecules obtained by merging pharmacophores are not necessarily effective. Secondly, most of the potent VEGFR inhibitors in clinical studies are multi-targeted, such as compounds **1**–**12**. It is noteworthy that these drugs are limited to a certain extent due to poor selectivity, potential toxicity or low metabolic stability, which seriously affects their clinical application. Thus, there is a pressing need to develop highly selective VEGFR inhibitors. Although highly potent and selective single-target drugs can temporarily solve these problems, these drugs are limited by drug resistance caused by the activation of compensatory signaling pathways. A superior approach is to identify dual-target VEGFR inhibitors with favorable selectivity and dual inhibitory potency that simultaneously inhibit at least two synergistic targets.

Reassuringly, besides the traditional drug discovery strategies described above, a number of novel approaches have been used for rational and efficient drug design of dual-function inhibitors. Particularly, computation-based approaches provide an opportunity to develop new dual-target VEGFR inhibitors. These strategies promote the identification of potentially rational target combinations of dual-target VEGFR inhibitors via predicting structural similarity between active sites of VEGFR and other tumor-related targets or reliable analyses of relevant signaling pathways. Additionally, structure- and ligand-based drug designs (SBDD and LBDD) have been widely applied in the development of dual-target lead compounds containing novel scaffolds and the molecular optimization of dual-target inhibitors [151]. Notably, artificial intelligence (AI) is an emerging trend in drug discovery. With the advanced development of technologies of AI, multiple approaches such as high-quality datasets, new hypotheses and machine learning models, and new algorithms have been developed and applied in the identification of dual-target VEGFR inhibitors [152]. Finally, the field of structural biology has encountered numerous technological breakthroughs. Consequently, a number of high-resolution structures of ligand–protein complexes have recently been obtained and provide a comprehensive overview of the molecular mechanisms of ligand–protein interactions. These findings afford insight into the structural modification via structure-based drug discovery and provide a structural basis for the identification of dual-target inhibitors.

Collectively, we highlighted the progress made in the development of dual-target VEGFR inhibitors to assess the physiological functions and morbid implications of relevant targets and discussed challenges and future directions in the discovery and rational design of more potent dual-target inhibitors.

## Data Availability

The material supporting the conclusion of this review has been included within the article.

## Abbreviations

AI: Artificial intelligence
AKT: Protein kinase B
BC: Breast cancer
BRAF: B-rapidly accelerated fibrosarcoma
DDIs: Drug–drug interactions
EGFR: Epidermal growth factor receptor
ERα: Estrogen receptor alpha
ERK1/2: Extracellular signal-regulated kinase ½
FDA: Food and Drug Administration
FGFR: Fibroblast growth factor receptor
HDAC: Histone deacetylase
LBDD: Ligand-based drug design
PARP: Poly ADP-ribose polymerase
PDGFR: Platelet-derived growth factor receptor
PIM1: Recombinant Pim-1 oncogene
SAR: Structure–activity relationship
SERM: Estrogen receptor modulator
TNBC: Triple-negative breast cancer
